# The evolving understanding of systemic mechanisms in organ-specific IgA nephropathy: a focus on gut-kidney crosstalk

**DOI:** 10.7150/thno.104631

**Published:** 2025-01-01

**Authors:** Xin Wang, Xu-Jie Zhou, Xue Qiao, Mario Falchi, Jing Liu, Hong Zhang

**Affiliations:** 1 Renal Division, Peking University First Hospital, Beijing, China.; 2 Peking University Institute of Nephrology, Beijing, China.; 3 Key Laboratory of Renal Disease, Ministry of Health of China, Beijing, China.; 4 Key Laboratory of Chronic Kidney Disease Prevention and Treatment (Peking University), Ministry of Education, Beijing, China.; 5 State Key Laboratory of Vascular Homeostasis and Remodeling, Peking University, Beijing, China.; 6 State Key Laboratory of Natural and Biomimetic Drugs, School of Pharmaceutical Sciences, Peking University, Beijing, China.; 7 Department of Twin Research and Genetic Epidemiology, King's College London, London, UK.; 8 CAS Key Laboratory for Biomedical Effects of Nanomaterials and Nanosafety and CAS Center for Excellence in Nanoscience, National Center for Nanoscience and Technology of China, University of Chinese Academy of Science, Beijing, 100190, China.

**Keywords:** IgA nephropathy, Gut-kidney crosstalk, Gut microbiota, Microbial metabolites, Mucosal immunology

## Abstract

The interplay between multiple organs, known as inter-organ crosstalk, represents a complex and essential research domain in understanding the mechanisms and therapies for kidney diseases. The kidneys not only interact pathologically with many other organs but also communicate with other systems through various signaling pathways. It is of paramount importance to comprehend these mechanisms for the development of more efficient therapeutic strategies. Despite extensive research in IgA nephropathy (IgAN), the most common kidney disease, the elaboration mechanism of IgAN remains challenging. Numerous studies suggest that alterations in the intestinal microbiome and its metabolites are pivotal in the progression of IgAN, opening new avenues for understanding its mechanisms. Interestingly, certain presumed probiotics, such as *Akkermansia muciniphila*, have been implicated in the onset of IgAN, making the exploration of gut microbiota in the context of IgAN pathogenesis even more intriguing. In this review, we summarize the status of gut microbiology studies of IgAN and explore the possible mechanisms and intervention prospects. Future research and treatment directions may increasingly emphasize systemic, multi-organ combined interventions to decelerate the advancement of kidney disease and enhance the overall prognosis of patients.

## 1. Introduction

Chronic kidney disease (CKD) is defined by kidney damage or a reduced kidney filtration function with an estimated glomerular filtration rate (eGFR) below 60 mL/(min·1.73m²) for more than three months 1. CKD is a progressive disease with no cure, with an estimated global prevalence of 9.1% and 4.6% of deaths annually attributable to impaired kidney function 2. It often appears together with other comorbidities, including cardiovascular, cerebrovascular and liver complications 3 and, if left untreated, progresses to end-stage kidney disease (ESKD). IgA nephropathy (IgAN) is the most common pattern of primary glomerular disease worldwide and a significant cause of CKD and kidney failure worldwide 4. More recent data suggested that half of the patients would progress to ESKD within ten years after kidney biopsy, and nearly 100% of patients were at risk of progression to kidney failure within their expected lifetime 5. It suggests an unclear understanding of the disease's pathogenic mechanisms, and the inadequacy of existing treatment strategies highlight the unmet clinical demands.

IgAN is characterized by galactose-deficient IgA1 (Gd-IgA1) deposition in glomerular mesangium associated with mucosal immune disorders 6. The classic manifestation of gross hematuria occurs concurrently with mucosal infection 7, thus suggesting aberrant mucosal immune responses and demonstrating the non-negligible importance of environmental factors in its development and progression.

The microenvironment of the human body contains a microbial community, defined as the microbiome, which includes bacteria, fungi, viruses, etc 8. The gut microbiota consists mainly of bacteria, particularly *Firmicutes*, *Bacteroidetes*, *Actinobacteria*, *Proteobacteria* and *Verrucomicrobia* 9. The immune system and the gut microbial ecosystem have been increasingly acknowledged as inter-organ crosstalk central effectors in health and disease. The gut microbiota plays a significant role in various aspects of human health, including digestion, immune function, metabolism and mental well-being. Studies reveal links between dysbiosis in the gut microbiota and diseases that affect the gut and organs like the brain, liver and kidneys 10. The crosstalk between the gut microbiota and distal organs is increasingly recognized, and host-microbiome interactions are being delineated.

Under physiological conditions, intestinal absorption ensures the uptake of beneficial microbial metabolites, whereas the kidneys maintain homeostasis by excreting potentially toxic metabolic end-products. Conversely, kidney failure results in the accumulation of gut microbiota-derived metabolites. Discharging these substances in the gut changes the intestinal microenvironment. It contributes to intestinal dysbiosis that adversely affects the inflammatory, endocrine and neurological pathways involved in CKD onset and progression and may impair multiple organs. A deeper understanding of the gut-kidney axis is essential to intervene in the network of mechanisms that connect various organs.

Elucidating the changes in gut microbes and their metabolites in CKD, particularly in conditions like IgAN, holds significant relevance for future interventions in multi-organ networks. Evidence from clinical studies suggests that both composition of gut microbiota and functional potential were altered in IgAN. Experimental animal models indicate that exposure to commensal or pathogenic bacteria may produce excessive abnormally glycosylated IgA in mucosa-associated lymphoid tissue (MALT). This review aims to provide up-to-date information on the gut microbiota and metabolites to establish a link between alterations to microbial composition, bacterially-derived metabolites and the possible mechanisms that trigger the onset of IgAN—additionally, some novel insights related to translational research.

## 2. Multi-organ crosstalk with chronic kidney disease

In the context of multi-organ interactions, inflammation and fibrosis represent prevalent pathological processes. By suppressing systemic inflammatory responses or selectively targeting inflammatory signaling pathways that affect multiple organs (such as NF-κB, TGF-β, etc.), protective effects can be exerted in patients with kidney disease, thereby slowing disease progression 11.

Metabolic disruption frequently emerges as a pivotal concern within the interplay of cardiac and renal functions. Mitigating the burden on the kidneys and other organs can be achieved through the regulation of metabolic pathways, including lipid metabolism, glucose metabolism, and oxidative stress. Notably, sodium-glucose co-transporter 2 (SGLT2) inhibitors not only modulate renal glucose reabsorption but also demonstrate protective effects in the heart and liver 12. The modulation of the gut-kidney axis through probiotics, prebiotics, or fecal microbiota transplantation (FMT) can reduce the production of harmful metabolites and lower the risk of renal injury.

**Figure 1:**
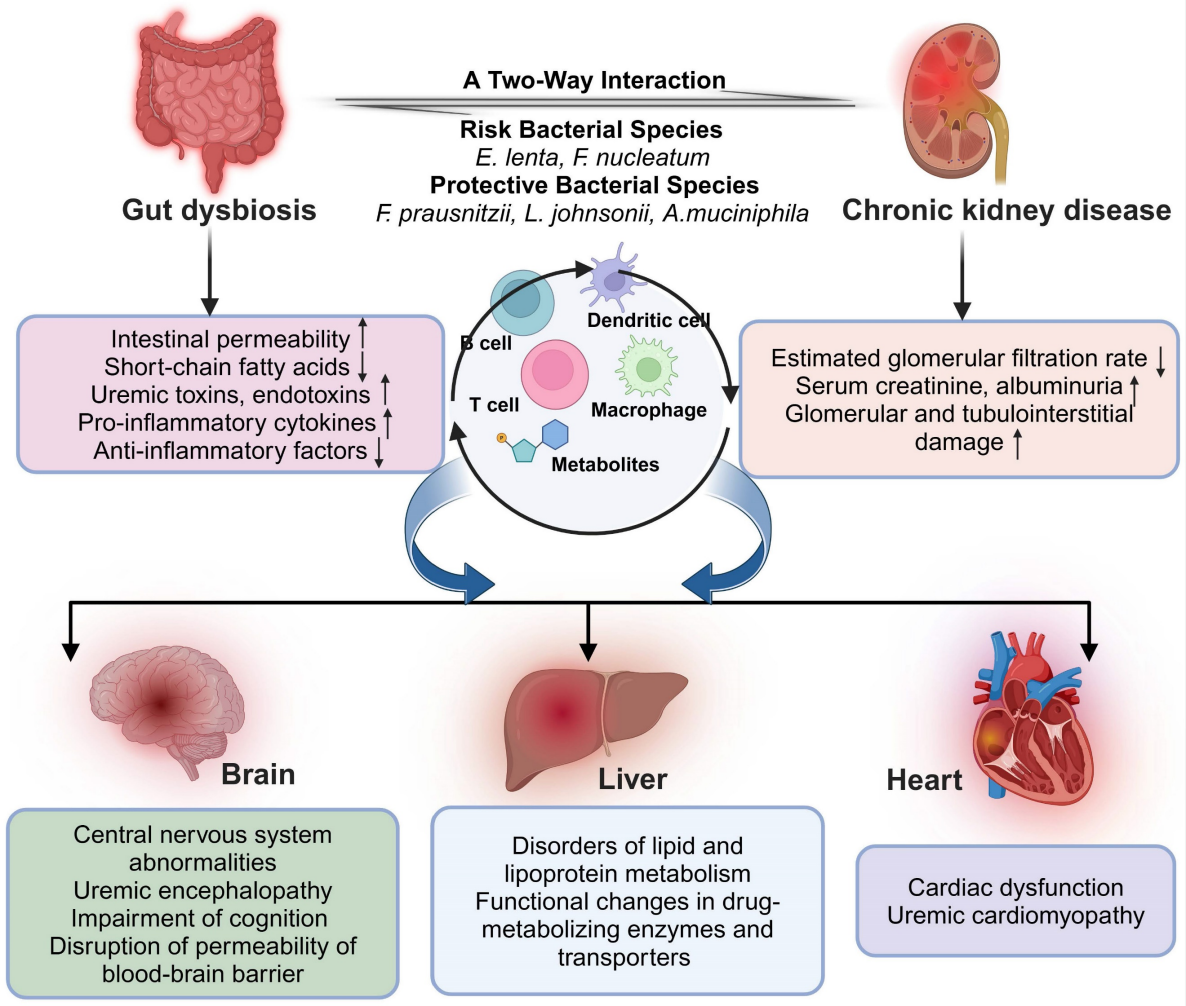
Illustration of Gut-kidney axis mediated specific organ cross-talk in Chronic Kidney Disease. Communication between the gut microbiota and its host in chronic kidney disease takes place across the multiorgan axis, with metabolites, interleukins, hormones, and toxins playing pivotal roles in mediating this interaction. Created with BioRender.com.

Understanding the complex interplay of multiple organs in renal disease reveals a sophisticated network of interactions, underscoring the importance of elucidating these mechanisms for the advancement of more efficacious treatment strategies. Future research and therapeutic approaches may lean towards systemic, multi-organ coordinated interventions to decelerate renal disease progression and improve patient outcomes. **Figure 1** illustrates the crosstalk between the renal dysfunction and the specific distal organs.

### 2.1. Gut-kidney axis in CKD

Changes in the gut microbiome are common in individuals with kidney disease, causing an increase in intestinal permeability. Consequently, bacterial metabolites from the gut, such as uremic toxins, are able to circulate through the bloodstream, exerting toxic effects on the kidneys. Conversely, impaired kidney function results in toxin buildup, further worsening intestinal dysfunction.

The emergence of 16S rRNA and metagenomic sequencing highlights the gut microbiota as an integral part of the microenvironment influencing CKD progression. Zhang and colleagues 13 analyzed 980 samples from six studies conducted in three countries. A notable decrease in gut microbiota diversity was observed in individuals with CKD when compared to those who are healthy. Nine species, *Ruminococcus gnavus*, *Ruminococcus bromii*, *Bacteroides fragilis*, *Alistipes onderdonkii*, *Bacteroides distasonis*, *Ruminococcus torques*, *Akkermansia muciniphila*, *Clostridium citroniae*, and *Bacteroides caccae*, were found to be significantly enriched in CKD patients. In contrast, six species, including *Blautia producta*, *Ruminococcus obeum*, *Coprococcus eutactus*, *Bacteroides plebeius*, *Prevotella copri*, and *Faecalibacterium prausnitzii*, showed a marked reduction in CKD. Alterations in the gut microbiota of individuals with CKD may vary across different disease stages or be limited to a specific stage. Wu *et al.* 14 conducted shotgun metagenome sequencing on 92 fecal samples to investigate alterations in species abundance during the progression of CKD. A decrease in four species (*Prevotella sp. 885, Weissella confuse, Roseburia faecis, and Bacteroides eggerthii*) and an increase in three species (*Alloscardovia omnicolens, Merdibacter massiliensis, and Clostridium glycyrrhizinilyticum*) was associated with CKD progression. Additionally, certain species were altered only at specific stages, including *Cetobacterium somerae* and *Candidatus Stoquefichus sp. KLE1796* in stage 1 and 2 CKD, *Fusobacterium mortiferum*, *Bariatricus massiliensis*, and *Bacteroides stercorirosoris* in CKD stage 3, and *Merdimonas faecis* in CKD stage 4 and 5. The findings imply that alterations in gut microbiota among CKD patients may be dynamic and stage-dependent. In the following, we explore the pivotal gut bacterial species within the microbiota and outline their roles, whether protective or detrimental, in the onset, evolution, and advancement of CKD.

#### CKD-risk bacterial species

The intestinal microbiota drives renal failure, at least in part via uraemic toxins. Wang *et al.* 15 found that ESKD-enriched species such as *Eggerthella lenta*, *Fusobacterium nucleatum*, and *Alistipes shahii* increased the production of uraemic toxins through aromatic amino acid degradation, secondary bile acids, and trimethylamine-N-oxide (TMAO) biosynthesis in the gut, resulting in higher levels of uraemic toxins and secondary bile acids in both faeces and blood of patients. Furthermore, rats subjected to gavage with *E. lenta* or *F. nucleatum* in a 5/6 nephrectomy (5/6Nx) CKD rat model exhibited higher serum levels of uraemic toxins when contrasted with sham-fed rats. This elevation was linked to increased oxidative stress, glomerulosclerosis, renal fibrosis, and elevated serum concentrations of creatinine and urea.

Dysbiosis in the intestinal microbiota has the potential to exacerbate complications of CKD. Hao *et al.* 16 analyzed fecal samples from CKD patients and rats with vascular calcification (VC) and revealed a notable elevation in *P. copri* compared to non-affected individuals. Oral administration of live *P. copri* aggravated CKD-related VC and osteogenic differentiation of vascular smooth muscle cells *in vivo*, accompanied by intestinal destruction, enhanced Toll-like receptor-4 (TLR4) expression, and elevated lipopolysaccharide levels in CKD rats. *P. copri* colonization alone did not induce aortic calcification in the absence of CKD, suggesting that the increased presence of *P. copri* could potentially contribute to vascular calcification associated with CKD.

#### CKD-protective bacterial species

***Faecalibacterium prausnitzii*** *Faecalibacterium prausnitzii*, a gram-positive anaerobic bacterium, exerts anti-inflammatory characteristics 17. Zhang *et al.* 13 revealed that the reduction in *F. prausnitzii* levels serves as a distinct microbial signature differentiating CKD patients from healthy controls (HCs). Li *et al.* 18 illustrated that supplementing the CKD mouse model with *F. prausnitzii* significantly reduced renal dysfunction, inflammation, and uremic toxins in the serum. This beneficial outcome is likely due to the anti-inflammatory effects of *F. prausnitzii* and its promotion of renal function through the production of butyrate.

***Bacteroides***, commensal gram-negative obligate anaerobes, play a vital role in colonizing the colon and constitute a significant portion of the gut microbiota. While some *Bacteroides* species can have beneficial effects in the gut, they may also exhibit opportunistic pathogenic behavior outside of the gastrointestinal tract 19. *B. fragilis* has been discovered in the gastrointestinal tract, the oral cavity, the upper respiratory tract and the female cervical tract 20. *B. fragilis* is classified into two main types based on the presence of a zinc-dependent metalloprotease known as the *B. fragilis* toxin. Strains that produce this toxin, enterotoxigenic *B. fragilis*, cleave E-cadherin, disrupting epithelial cell adhesion and inflammation 21. Strains lacking this toxin are considered nontoxigenic *B. fragilis* and potential probiotics. Zhou *et al.* 22 showed that oral administration of live *B. fragilis* reduces renal fibrosis in unilateral ureteral obstruction model and adenine mouse models by lowering lipopolysaccharide levels and increasing 1,5-anhydroglucitol. This mechanism alleviates renal fibrosis through the inhibition of oxidative stress and inflammation. *Bacteroides plebeians*, a bacterium residing in the human gut and commonly found in individuals with a seaweed-rich diet, harbors a polysaccharide utilization locus that selectively degrades porphyrin and agarose from red seaweeds 23. Pei *et al.* 24 observed that administering *B. plebeius* orally effectively prevented muscle wasting in rats following 5/6Nx, acting through the Mystn/ActRIIB/SMAD2 pathway.

***Lactobacillus johnsonii*** *Lactobacillus johnsonii*, a Gram-positive bacterium, is probiotic bacterial species with broad antimicrobial properties 25. Miao *et al.* 26 found that oral *L. johnsonii* attenuated renal fibrosis by suppressing Aryl hydrocarbon receptor (AHR) signal via increasing serum indole-3-aldehyde.

***Akkermansia muciniphila*** *Akkermansia muciniphila*, a bacterium that degrades mucin, can inhabit the intestines of mammals like humans and mice by utilizing mucin as its sole nitrogen and carbon source 27. Extensive research has been conducted on the probiotic properties of *A. muciniphila*, encompassing metabolic regulation, immune modulation, and safeguarding gut health 28. The study conducted by Pei *et al.* unveiled that *A. muciniphila* possesses the ability to restore disrupted gut microbiota, reinforce the intestinal mucosal barrier, diminish inflammation, and alleviate interstitial fibrosis in rats with CKD 29. However, the onset of IgAN was observed in mice modified to express human IgA1 and the human Fc alpha receptor I following colonization with *A. muciniphila*, as reported by Gleeson *et al.* 30.

Apart from bacteria, alterations have been reported in the fecal virome, including phages, in patients with diabetic nephropathy (DN) 31. A study involving 90 subjects with or without type 2 diabetes (T2D) and 42 HCs from China found changes in gut viral diversity are more prominent in T2D with nephropathy compared with T2D without DN. At the species level, 14 viral species were identified to be associated with DN, of which 85% belonged to phages. Of these, 12 species (e.g., *Bacteroides phage* and *Anoxybacillus virus*) were significantly decreased, whereas two species (*Shigella phage* and *Xylella phage*) were increased in DN. Moreover, six species were identified as differential markers only in T2D with nephropathy, including *Erysipelothrix phage*, *Lactococcus phage*, *Faecalibacterium virus*, *Brevibacillus phage*, *Bacteroides phage*, and *crAssphage cr114_1*. In addition, significant positive correlations of viral richness and bacterial diversity were observed in T2D and T2D with nephropathy. These results suggest that DN subjects have significant gut viral disturbances and the presence of virus-bacteria interactions. However, the causal relationship between the phage and bacteria is still unclear, and further studies on the underlying mechanisms are essential for identifying potential therapeutic targets in CKD including DN.

The Epstein-Barr (EB) virus induces subclinical infection in a significant proportion of individuals. It has been established that when B cells are infected with this virus, they generate Gd-IgA1 32. Recent findings indicate a marked increase in EB virus-infected plasma cells/plasmablasts among IgAN patients, despite no variation in the distribution of each B-cell subset among CD19-positive cells in peripheral blood compared to individuals with other forms of nephritis and those who are healthy 33. Large scale virome study has not yet been reported in IgAN.

Fungal members of microbial communities on mucosal surfaces are part of our bodies' normal ecology. Using ITS ribosomal RNA gene sequencing, Hu *et al.* 34 observed that CKD had more *Saccharomyces* and lower levels of *Candida*, *Bjerkandera*, *Rhodotorula*, and *Ganoderma* than HCs. The influences of gut fungi on CKD were investigated using oral* Candida albicans*-administered 5/6Nx mice 35. It was found that the *Candida*-5/6Nx mice mouse had a higher abundance of *Proteobacteria*, *Helicobacter spp.* and *Allobaculum spp.* and more severe gut leakage with higher serum glycaemia and increased serum cytokines than non-*Candida*-5/6Nx 35. Dysbiosis of gut fungi may affect the function of caspase recruitment domain-containing protein 9 (CARD9), a susceptibility gene for autoimmune glomerulonephritis including IgAN, thereby the activation of inflammatory immunity and interleukin-17A-producing T helper cell, contributing to the development of CKD 36. Due to limited numbers of studies, the spectra of gut fungi in CKD remain unclear.

### 2.2. Gut-kidney and brain axis

The Brain-Gut-Kidney Axis represents a burgeoning field of research into the interplay among multiple organ systems. This axis elucidates the intricate physiological and pathological connections between the nervous system, gastrointestinal system, and kidneys, particularly highlighting the profound impact of their interactions on human health in chronic diseases and metabolic disorders 37. The autonomic nervous system's sympathetic and parasympathetic branches regulate intestinal motility, secretion, and barrier function via the vagus and spinal nerves. Conversely, the gut microbiota and their metabolic byproducts can impact the function and behavior of the central nervous system 38. The kidneys impact brain function by regulating blood pressure, eliminating metabolic waste, and preserving fluid balance. Simultaneously, the brain influences kidney function through neural and endocrine signaling pathways like the renin-angiotensin and sympathetic nervous systems. CKD frequently disrupts brain function due to toxin buildup, manifesting as cognitive impairment and disrupted sleep patterns 39.

As renal function deteriorates, detrimental metabolic byproducts, including uremic toxins, accumulate. These substances reach the brain via the bloodstream, causing neurological abnormalities 40. Moreover, they disrupt gut microbiota balance. Inflammatory reactions and toxin generation in the gut worsen the condition and may also affect brain inflammation and cognitive function, perpetuating a detrimental cycle. Gut microbial products like Indoxyl sulfate (IS), obtained after the metabolism of amino acids, are harmful to the brain. Rats fed an adenine-rich diet with drinking water containing IS showed increased serum concentration of IS impairment of cognition and increased blood-brain barrier permeability 41. IS also induces apoptosis of astrocytes via oxidative stress and protein kinase inhibition 42. Moreover, blood-brain barrier damage in uremic patients may result from disrupting endothelial tight-junction proteins 43. Some mendelian randomization (MR) analysis also revealed a causal link between kidney damage and alterations in cortical brain structure, supporting causal evidence of the kidney-brain axis 44.

Alterations in the gut microbiota are closely associated with various neurological disorders, including Parkinson's and Alzheimer's diseases, which are often accompanied by renal impairment 45. The degeneration of the nervous system can disrupt renal function by modulating the autonomic nervous system, resulting in electrolyte imbalance and subsequently kidney function. Additionally, neurodegeneration impacts gut function, leading to complications such as constipation and intestinal barrier dysfunction, which in turn affect the metabolic workload on the kidneys. Systemic inflammation can occur in the case of ESKD, leading to the translocation of bacteria and their products into systemic circulation, which help to activate the immune response. Systemic inflammation may activate resident macrophages called microglial cells in the central nervous system 46.

### 2.3. Gut-kidney and liver axis

The liver and intestines are intricately linked through the portal vein system. Nutrients, microbial metabolites, and toxins absorbed by the intestines enter the liver for processing via the portal vein. The interplay between the liver and kidneys is mainly mediated through systemic metabolism, toxin elimination, and hemodynamic mechanisms. Dysbiosis of gut microbiota and heightened intestinal permeability can cause bacterial metabolites (e.g., lipopolysaccharides, LPS) and toxins to enter the bloodstream, prompting an inflammatory reaction in the liver, potentially leading to liver fibrosis or cirrhosis and further compromising renal function via systemic inflammatory responses 47. Simultaneously, impaired liver function, as seen in conditions like cirrhosis, can lead to intracellular water and sodium retention, subsequently activating the renin-angiotensin-aldosterone system in the kidneys, causing renal dysfunction such as reduced renal blood flow, ultimately culminating in hepato-renal syndrome 48. In patients with mild to moderate CKD, lipid and lipoprotein metabolism alterations are evident, characterized by hypercholesterolemia and elevated low-density lipoprotein cholesterol levels, particularly notable in those with nephrotic proteinuria 49. CKD induces changes in lipoprotein composition, partly attributable to the uremic milieu, which fosters a broader molecular diversity of lipoproteins and irreversible post-translational modifications due to compromised renal function 50.

The interaction within the gut-kidney-liver axis becomes particularly significant in the context of drug transporters. Functional changes in these transporters and drug-metabolizing enzymes can be attributed to the inhibitory impact of uremic toxins and the influence of inflammatory cytokines 51. Rosenthal *et al.* 52 identified that the chosen group of ATP-binding cassette transporters, solute carriers and drug-metabolizing enzymes form the most substantial gut-liver-kidney cluster of inter-connected genes among a random network of 690 genes. Uremic toxins are suggested to regulate the AHR. IS has been shown to regulate hepatic P-glycoprotein via AHR in rodent and cell models 53. Clinical studies have also shown a correlation between high P-glycoprotein expression levels in CKD and elevated plasma IS levels. This could potentially impact the hepatic metabolism of drugs such as cyclosporine 54. In patients with CKD, uremic toxins can also potentially inhibit and downregulate hepatic pharmacokinetic proteins, including organic anion-transporting polypeptide-1B, cytochrome P450 and UDP-glucuronosyltransferase 55. The gut microbiome produces trimethylamine through choline metabolism, which is converted in the liver to TMAO by flavin-containing monooxygenases 56. TMAO has been implicated in suppressing bile acid-mediated farnesoid X receptor signaling in the liver, potentially exacerbating liver steatosis 57. Additionally, proinflammatory cytokines, such as interleukin (IL)-6, have been positively associated with CKD severity and are known to transcriptionally reduce the expression levels of P450 enzymes 58.

### 2.4. Gut-kidney and heart axis

The prevalence of cardiovascular disease (CVD) is markedly higher among individuals with CKD compared with those without CKD 59. Patho-physiologically, CKD and CVD patients are prone to gastrointestinal dysfunction and intestinal microecology disorder. Chronic inflammation and reactive oxygen species generation, often triggered by pathogenic bacteria or their endotoxins, are implicated in this gut-kidney-heart axis 60. The relationship between the heart and the intestines is demonstrated by the influence of cardiovascular diseases on the intestinal microenvironment, through alterations in blood flow dynamics and metabolism. Diminished cardiac function reduces blood perfusion, impacting intestinal oxygen delivery and leading to intestinal hypoxia and subsequent barrier compromise. This barrier dysfunction enables bacterial metabolites to enter the bloodstream, inciting systemic inflammation that, in turn, affects heart function, in a detrimental cycle 61.

Hypertension represents a key risk factor for renal diseases, with excessive activation of the sympathetic nervous system in the brain frequently identified as a causal factor 62. Additionally, gut microbiota dysbiosis is considered a contributing factor in the development of hypertension. Metabolites from the gut microbiota, including short-chain fatty acids (SCFAs) and bile acids, indirectly regulate renal and blood pressure control by impacting vascular tone, inflammation, and immune responses 63.

Bacterial endotoxin, a LPS constituent of the external cell wall of most gram-negative bacteria, is continuously produced in the gut and translocated into the systemic circulation across the intestinal barrier 64. Observational studies have highlighted significant correlations between circulating bacterial DNA levels, serum C-reactive protein and IL-6 levels, and the risk of CVD events in patients with ESKD 65, 66. Experimental studies suggest circulating bacterial DNA fragments can directly impact the cardiovascular system, notably by suppressing cardiac myocyte contraction 67.

TMAO concentrations have been related to atherosclerosis. Higher TMAO levels and pro-inflammatory cytokine expression are observed to accompany cardiac dysfunction in mouse models. *Klebsiella pneumoniae* enriched in CKD would contribute to developing uremic cardiomyopathy via the induction of heart-infiltrating IFNγ^+^ CD4^+^ T cell expansion 68. Furthermore, the gut microbiota regulates vitamin D metabolism through fibroblast growth factor 23. The α-Klotho protein, the receptor for fibroblast growth factor 23, is mainly expressed in the kidney, parathyroid gland, and choroid plexus and is significantly reduced in CKD, a condition associated with profound cardiovascular dysfunction 69. The comprehension of this axis presents a renewed viewpoint on the prevention and treatment of heart and kidney ailments and the management of associated metabolic syndromes.

## 3. Microbiota, mucosal immunity and Gd-IgA1

One of the main antibodies in the immune system, IgA, is primarily localized in the mucosal system, specifically within the intestinal tract. B cells in the gut secrete IgA to counteract pathogens and exogenous antigens in the intestinal milieu. The gut microbiota normally upholds the equilibrium of mucosal immunity, preventing the elicitation of abnormal immune reactions by the IgA antibodies produced 70. Nevertheless, dysbiosis in the gut microbiota can disrupt this balance, leading to abnormal IgA production, formation of abnormal immune complexes, their systemic dissemination, and subsequent deposition in the renal glomeruli, culminating in IgAN.

The modulation of mucosal immune responses and IgA production by the gut microbiota is crucial. Dysbiosis in the gut microbiota, particularly a decrease in probiotics and an increase in opportunistic pathogens, has been observed in patients with IgAN. These microbial imbalances may influence the development and advancement of IgAN through various mechanisms:

(1) Dysregulation of mucosal immunity: dysbiosis in the gut microbiota can compromise intestinal barrier function, allowing bacteria and toxins to translocate across the intestinal wall, thereby activating the gut immune system and leading to excessive IgA production.

(2) Disruption in intestinal homeostasis may impact the structure and function of IgA, leading to the formation of pathologically significant IgA immune complexes that enter the bloodstream and deposit in the kidneys.

(3) Enhanced inflammatory signals: dysbiosis in the gut microbiota can lead to increased release of pro-inflammatory cytokines by intestinal epithelial cells, triggering systemic immune responses and exacerbating renal inflammation.

**Figure 2:**
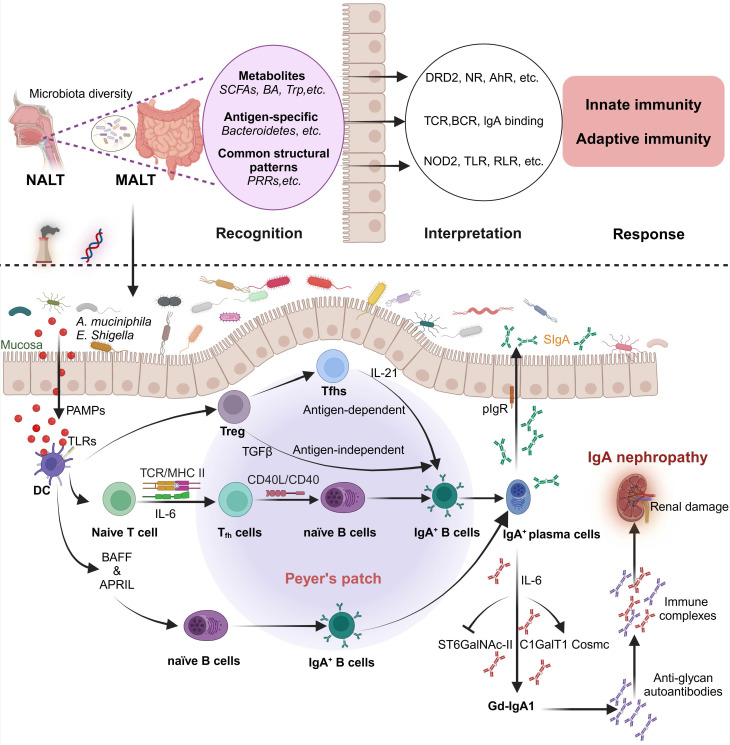
The potential mechanism of Gd-IgA1 production in a multi-hit model of IgA nephropathy. Microbial colonization represents a conditioning exposure that directs functional maturation of host innate and adaptive immunity through the actions of metabolites, foreign molecular patterns and antigens. Microbiota-derived metabolites trigger chemosensory receptors. For example, activation of dopamine receptor D2 (DRD2) in the intestinal epithelium by gut microbial metabolism of L-tryptophan (L-Trp), particularly through the production of indole derivatives, confers protection against *Citrobacter rodentium*, a mouse model for enterohemorrhagic *Escherichia coli* infection 197. Microbial bile acid (BA) metabolites regulate gut RORγ^+^ regulatory T cell homeostasis and ameliorate host immunologic homeostasis through BA nuclear receptors (NR) in mice 198. Microbiome-derived antigens and immunomodulatory signals have also documented the conditioning of adaptive immunity. For instance, in the T cell receptor (TCR) transgenic model that was specific for *Bacteroidetes spp*., adoptive transfer of transgenic T cells suppressed colitis induced by co-transfer with naive CD4^+^ T cells, and this effect was recognized by CD4^+^ intraepithelial lymphocytes 199. Additionally, the microbiome also conditions the innate immune system via conserved molecular patterns directly recognized by pattern recognition receptors. Firmicutes-derived DL-endopeptidase protects mice from colitis through activation of nucleotide oligomerization domain 2 (NOD2) 200. An unhealthy lifestyle due to increased and sustained stress, infection, or other factors can cause gut dysbiosis. The Gd-IgA1 may be produced and regulated by gut microbiome via crosstalk of the T-cell-dependent and/or the T-cell-independent pathway in IgA nephropathy. Abbreviations: AhR: Aryl hydrocarbon receptor; APRIL: a proliferation-inducing ligand; BAFF: B cell activation factor of the TNF family; BCR:B cell receptor; DC: dendritic cell; Gd-IgA1: galactose-deficient IgA1; GALT: gut-associated lymphoid tissues; NALT: nasal-associated lymphoid tissue; PAMPs: pathogen-associated molecular patterns; PRRs: pattern recognition receptors; RLR: rig-I-like receptor; SCFAs: short-chain fatty acids; TLR: toll-like receptor; Tregs: regulatory T cells; Tfh: t follicular helper. Created with BioRender.com.

A critical factor in the pathogenesis of IgAN is the dysregulation of the glycosylation of IgA molecules, particularly affecting the highly glycosylated IgA1 subclass characterized by the presence of galactose-deficient O-glycans in the hinge region of IgA1. Glycosylation is a post-translational modification that enhances antibodies' conformational diversity, affecting immunoglobulins' structure, form and effector functions 71. The precise source and stimuli for producing pathogenic IgA are unknown. A widely accepted hypothesis for the pathogenesis of IgAN is a multi-hit model. In this model, Gd-IgA1 is present in circulation at elevated levels in patients with IgAN (hit1). This immunoglobulin is recognized by unique circulating anti-glycan autoantibodies (hit 2). This process results in the formation of pathogenic immune complexes (hit 3). Finally, the immune complexes are deposited in the glomerular mesangium and induce renal damage (hit 4). Since Gd-IgA1 is a critical molecule in its pathogenesis, elucidation of the formation of Gd-IgA1, such as nasal-associated lymphoid tissue (NALT) and gut-associated lymphoid tissues (GALT), is crucial to understanding disease processes. The production of Gd-IgA1 in a multi-hit model is summarized in** Figure 2**. It is hypothesized that genetic predisposition to mucosal infection and concomitant IL-6 production can lead to aberrant glycosylation by modifying the glycosylation machinery 72, 73.

The genome-wide association studies (GWAS) of IgAN have shown that Gd-IgA1 levels are highly heritable (estimated at 54%-80%) 74. Two quantitative trait GWAS for Gd-IgA1 levels have identified two genome-wide significant loci, in *C1GALT1* and *C1GALT1C1*, influence Gd-IgA1 level in the population, which independently associates with risk of progressive IgAN 75, 76. Our study discovered that a novel locus, *GALNT12*, exhibits genetic interactions with *C1GALT1* in Gd-IgA1 levels and disease risk 77. Recent studies indicated that C1galt1 deficiency in mice results in changes in the intestinal microbiota and impaired mucus barrier function, enabling rapid breach of the mucus layer by bacteria 78, 79. Our previous study showed that the risk genotypes of *LYZL*1 affecting the gut microbiome and susceptibility to IgAN, were associated with higher serum levels of Gd-IgA1 80. Whether altered galactosylation processes result from immunometabolic signals emanating from gut microbiota remains unknown. A metagenomics-based analysis study from intestinal microbiota showed that α-galactosidase and α-N-acetyl-galactosaminidase secreted by *Flavonifractor plautii* may contribute to the production of Gd-IgA1 in IgAN 81. There were also studies showed that decreases in the levels of normal bacteria, such as members of the genera *Prevotella* and *Bifidobacterium*, were related to increased levels of Gd-IgA1 82 and increases in the levels of *Bacteroides* and *Parabacteroides* were positively correlated with serum Gd-IgA1 levels in IgAN 83.

In mucosa-associated lymphoid tissue, including NALT and GALT, the mucosal immune response can induce Gd-IgA1 production by peripheral B cells. The interaction of mucosally derived antigens with B cells includes activation through T-cell-dependent or T-cell-independent pathways. The latter involves the interaction between B cells, dendritic cells and the TLRs pathway. Persistent activation and overactivation of TLRs might induce the overproduction of Gd-IgA1 and autoantibodies. TLR9, the A proliferation-inducing ligand and IL-6-mediated pathways were suggested to be involved in synthesizing Gd-IgA1 72. Studies showed that the mechanisms of the IL-6-enhanced aberrant glycosylation of IgA1 involved dysregulated expression and activity of glycosyltransferases, including upregulation of ST6GalNAc-II, downregulation of C1GalT1 84 and overexpression of GalNAc-T14 73. This process is potentially triggered through the Jak2/STAT3 signal pathway 85. Additionally, signaling of the IL-6 family cytokines leukemia inhibitory factor (LIF) in the cells from IgAN patients might involve abnormal activation of the STAT1 pathway, contributing to the production of Gd-IgA1 86.

## 4. Microbiota in IgAN: evidence from clinical and experimental studies

### 4.1. Community composition of gut microbiota in IgAN: evidence from population association studies

**Figure 3:**
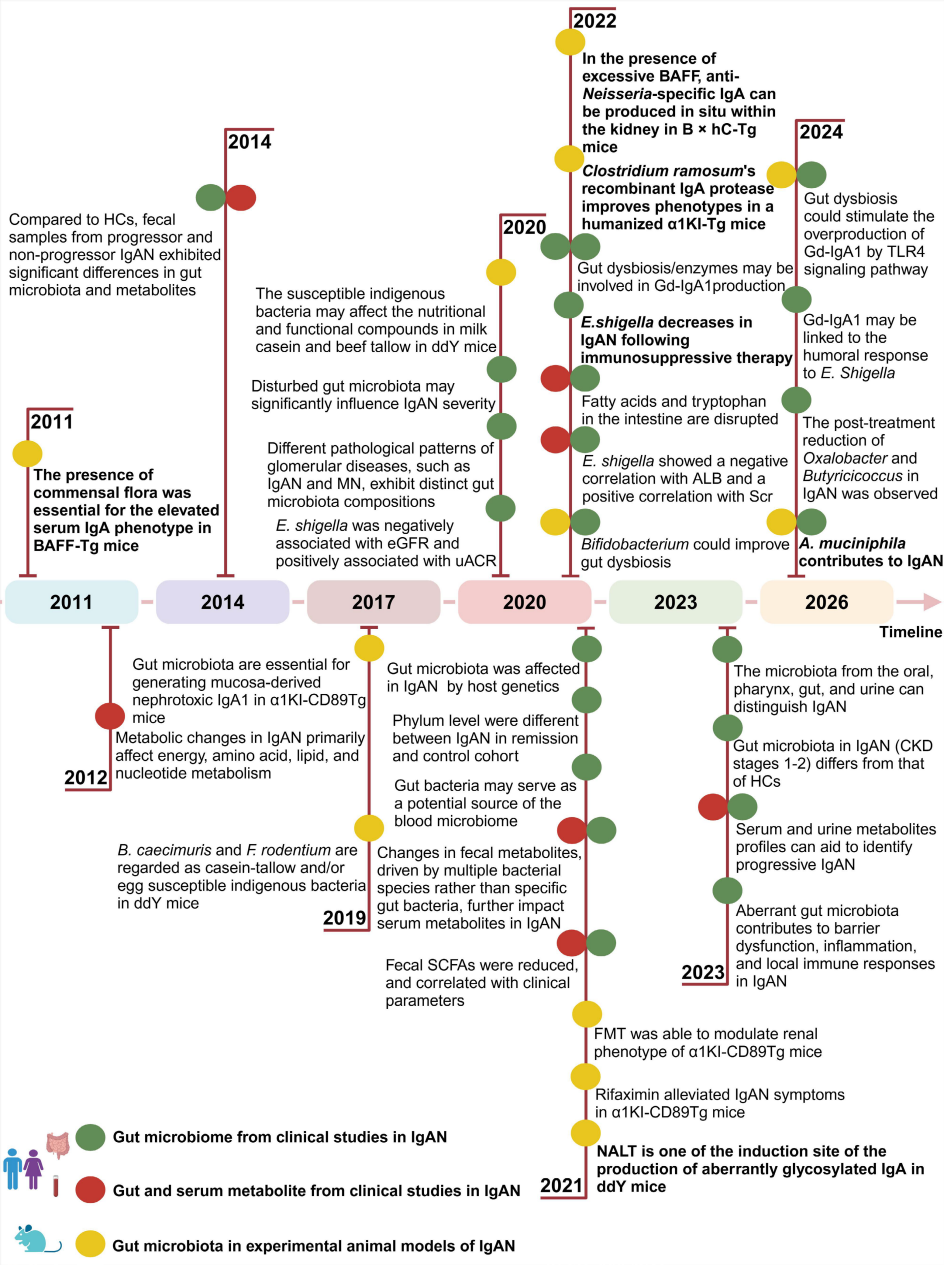
Timeline of gut microbiota and/or metabolomics studies in IgA nephropathy and experimental animal models. Abbreviations: ALB: albumin; BAFF: B cell activation factor of the TNF family; BAFF-Tg mice: BAFF overexpressing transgenic mice; eGFR: estimated glomerular filtration rate; uACR: urinary albumin-to-creatinine ratio; CKD: chronic kidney disease; FMT: fecal microbiota transplantation; Gd-IgA1: galactose-deficient IgA1; HCs: healthy controls; IgAN: IgA nephropathy; MN: Membranous nephropathy; NALT: nasal-associated lymphoid tissue; SCFAs: short-chain fatty acids; Scr: serum creatinine; TLR4: toll-like receptor 4; uACR: urinary albumin-to-creatinine ratio. Created with BioRender.com.

Over the past few decades, advancements in next-generation sequencing technology have played a crucial role in elucidating the intricate connection between the microbiome and various diseases. A high systemic antibody response, including a greater rate of a more pronounced IgA and IgG anti-*Helicobacter pylori* antibody response to mucosal infection caused by *Helicobacter pylori* in patients with IgAN, has been reported since year 2006 87. Our previous study showed that *Helicobacter pylori* infection was associated with elevated Gd-IgA1 in IgAN 88. A wealth of evidence supports the notion that IgAN is frequently accompanied by dysbiosis of the gut microbiota (**Figure 3**). Most of these studies were cross-sectional, except for one, and the majority took 16S rDNA sequencing for gut microbiome analysis. The findings of recent research on gut dysbiosis in individuals with IgAN are outlined in **Table 1**.

Twenty-four studies have been systemically reviewed and summarized. The studies included in the analysis were exclusively from Asia and Europe, with nineteen originating from China, one from Korea, one from Malaysia, two from France, and one from Italy.

The studies identified significant microbial variations, particularly observed at the genus level. Nonetheless, only a minor subset of gut microbiota consistently yielded congruent results across the diverse studies. Thirteen studies reported that proportion of* Escherichia-shigella* showed significantly higher levels in IgAN than in HCs 82, 83, 89-99. No study has yet reported that the level of *Escherichia-Shigella* decreased in IgAN. In these studies, many findings confirmed that a high abundance of *Escherichia-Shigella* generally correlates with elevated Gd-IgA1 levels. Zhao *et al.* 92 found seven microbial OTUs as optimal bacterial markers for distinguishing patients with IgAN from HCs, with *Escherichia-Shigella* contributing the most. Gao *et al.* 98 reported similar findings, who also observed a heightened IgA1 antibody response to *Escherichia-Shigella* and their main bacterial antigen stx2 in IgAN patients. Nine studies found that the relative abundance of the* Bacteroides* genus is higher in patients with IgAN compared to HCs 80-82, 94, 95, 98-101. Eight studies have reported that the relative abundance of the *Prevotella* genus is significantly reduced in IgAN 30, 80-82, 94, 98, 101, 102. Higher eGFR was associated with a greater abundance of *Prevotella* by Peters *et al.* 103.

The consistency of these studies underscores the potential critical involvement of *Escherichia-Shigella*, *Bacteroides*, and *Prevotella* in IgAN development. Yet, alterations in microbial family and genus proportions may not sufficiently capture microbiota changes, necessitating future investigations focusing on specific species or strains.

Although findings on other gut bacteria, such as *Akkermansia*, have been inconsistent across studies, their potential role in the progression of IgAN should not be underestimated. Gleeson *et al.* 30 demonstrated that *A. muciniphila* plays a pivotal role in the pathophysiology of IgAN. In mice that expressed human IgA1 and Fcα receptor I (α1KI-CD89tg mice), the quantity of deglycosylated IgA1 correlated with the relative abundance of *A. muciniphila* in the intestinal lumen. Further analyses revealed that IgA1 undergoes deglycosylation upon direct interaction with live bacteria in the intestinal lumen. This deglycosylation process promotes the translocation of IgA1 from the intestinal lumen to the circulation through retro-transcytosis. Moreover, human IgA1 incubated with *A. muciniphila* was identified by autoantibodies in the sera of IgAN patients. In α1KI-CD89Tg mice treated with broad-spectrum antibiotics to eliminate gut microbiota, reintroduction of *A. muciniphila* (but not *Escherichia coli*) resulted in exacerbated IgAN manifestations. It concluded that mucin-degrading bacteria are directly responsible for producing the deglycosylated IgA1 autoantigen in IgAN. In the future, various avenues must be investigated to unlock the therapeutic possibilities. These avenues include methods to boost the synthesis of α-defensins, which impede the proliferation of *A. muciniphila* on the mucosal surface, tactics to combat mucin-degrading bacteria and their enzymes that strip IgA1 of its glycans, and dietary interventions to alter the gut microbiota in individuals with IgAN.

Apart from susceptibility association, specific bacterial species displayed unique abundance patterns in IgAN non-progressors and progressors, underscoring the significance of gut microbiota in disease progression. De *et al.* 96 found that a higher proportion of *Bifidobacterium* had higher levels in non-progressor patients than in progressor. The abundance of *Prevotella* increased in progressor patients compared to non-progressor. The non-progressor patients with IgAN had a higher abundance of *Bacteroides coprocola*, *B.fragilis*, *Bacteroides vulgatus*, and a higher proportion of *Bacteroides finegoldii*, *Bacteroides intestinalis*, *B. plebeius* and *Bacteroides salyersiae* were richer in progressor patients with IgAN. However, due to sample size limitations and disease heterogeneity, care interpretation of the data and larger follow-up replications may be needed.

As mentioned above, despite the predominant focus of existing research on cataloging bacterial taxa, it is crucial to acknowledge the existence of other microorganisms, such as bacteriophages, in the gut. Studies have highlighted the significance of bacteriophages in influencing microbiota stability, with implications for altering microbiota composition, increasing intestinal permeability, and inciting persistent inflammation 104. The potential role of other neglected components of gut microbiota, also deserves further study for comprehensive understanding of aetiology and pathology of IgAN.

### 4.2 Functional potential of gut microbiome in IgAN: clinical association clues

Several human studies have employed “omics” techniques and thus added new perspectives on functional attributes of the gut microbiome in IgAN. The results of recent studies about fecal and serum metabolite in IgAN patients are listed in **Table 1.** The systemic changes in endogenous metabolites from IgAN mainly influenced fatty acid, amino acid, and nucleotide metabolism. For instance, compared to HCs, the levels of intestinal SCFAs, fatty acid, 3-indolepropionic acid in IgAN 102, 105, 106. The richness of species within the gut microbiome is closely associated with metabolic diversity. Notably, *Streptococcaceae* showed a positive correlation with both fecal and serum bilirubin levels. The increase in fecal metabolites, such as phenylalanine and bilirubin, correlates directly with their respective levels in the serum 102. It was shown that a marked increase of total FAA was found in the fecal samples of IgAN patients, and serum samples of IgAN patients also had a rise of some FAA (e.g., Asp, Glu and Tyr) 107.

Studies also have identified differences in metabolite profiles between non-progressor and progressor IgAN patients. For example, some metabolites (Acetone, Glycerol, Glycine, Threonine, Valine) increased in non-progressor patients with IgAN. In contrast, some metabolites (Formate, Betaine, N, N-Dimethylglycine) increased in progressor patients with IgAN 108. Some studies reported relevant correlations between metabolite alterations and IgAN clinical features. For example, high levels of Gd-IgA1 were associated with lower levels of 3-indolepropionic acid 106. Enriched catechol, azelaic acid, mandelic acid, and l-tryptophan were positively correlated with serum creatinine, uric acid, and 24 total urinary proteins and negatively correlated with eGFR 91. Despite being cross-sectional, the studies are still somewhat scarce, warranting more strong evidence from well-designed studies. The levels of metabolites are subject to great fluctuations and across different time and assays due to the interplay between microbiota, diet, environment, and medications.

### 4.3. Microbiota in IgAN: supporting evidence from model animals

A study 109 involving B cell activation factor of the TNF family (BAFF) overexpressing transgenic mice demonstrated that these mice develop IgA-driven nephritis contingent on commensal flora. This finding suggests that elevated levels of BAFF alone are insufficient to induce IgA-associated renal injury. However, through interactions with commensal flora, they contribute to an IgAN-like pathology. Some other studies also emphasized the pivotal role of gut microbiota in generating mucosal-derived nephrotoxic IgA1, promoting occurrence or progression of IgAN 110. This was particularly evident in FMT experiments in α1KI-CD89Tg mice models 111. Microbiota from patients with severe disease stages notably contributed to the IgAN phenotype in mice. It was further discovered that mice colonized by *A. muciniphila* developed an exacerbated IgAN phenotype in the α1KI-CD89Tg mouse model 30. Alterations in gut microbiota composition were observed in IgAN mice, with decreased levels of *Bifidobacterium* and *Lactobacillus* and increased percentages of *Helicobacter* and *Alloprevotella* 101. While rifaximin decreased IgAN symptoms in α1KI-CD89Tg mice, it remains unclear whether these results stem from modulation of the intestinal microbiota or other effects of rifaximin on the gut 112.

The initiation of IgAN in germ-free ddY mice also offered valuable perspectives 113. These mice did not present IgAN symptoms in a germ-free milieu but experienced heightened kidney damage featuring mesangial IgA accumulation upon transition to a specific pathogen-free environment. This observation underscores the significance of the NALT over the GALT in stimulating nephritic IgA synthesis in these specific mouse models. However, we may note that the absorption of oligodeoxynucleotides is generally sluggish, and its degradation may be a pertinent issue. Furthermore, indigenous gut bacteria in ddY mice were found to be responsive to specific dietary components, including *Bacteroides acidifaciens* and *Bacteroides caecimuris* (responsive to casein and beef tallow) and *Faecalibaculum rodentium* and *Allobaculum stercoricanis* (responsive to casein and egg powder) 114, 115. The data summarized in **Table 2** underscores the significance of microbiota composition in shaping the nephritogenic phenotype.

## 5. Potential mechanisms of gut microbiota in IgAN

Due to its multifactorial etiology of IgAN, a precise investigation of the pathogenesis is extremely difficult. It is essential to note that IgAN is a heterogeneous condition, with secondary forms potentially linked to viral hepatitis, IBD, and other conditions. Primary IgAN, on the other hand, shows associations with numerous genetic variants. Extrapolating data from animal models to patients also remains challenging due to differences in immune responses, especially on IgA glycosylation. However, judged to be promising, plenty of studies have outlined potential mechanisms through which gut microbiota may contribute to IgAN, influenced by factors such as diet and genetic predispositions shared with gastrointestinal disorders. As research deepens, we focus here on the potential mechanisms linking gut microbiota and IgAN, which can be updated into five perspectives in detail:

(1) Genetic susceptibility: host specific genetic backgrounds may increase the sensitivity of intestinal bacteria to IgAN, serving as a primary trigger for the development of IgAN.

(2) Epigenetic mediation: epigenetic modifications may serve as crucial mediators between the gut microbiota and IgA production.

(3) Impaired gut barrier: dysregulation of mucin-degrading bacteria disrupts the gut barrier, leading to abnormal glycosylation of IgA.

(4) Molecular mimicry and microbial metabolites: gut dysbiosis results in an imbalance of microbe-associated metabolites, impacting lymphocyte differentiation and cytokine production.

(5) B cell activation: intestinal dysbiosis can lead to aberrant activation and differentiation of IgA-producing B cells in the gut.

### 5.1. Gut microbiota: host genetic susceptibility background

Recent advances in understanding the etiological role of gut microbiota in IgAN have been significantly driven by insights garnered from GWAS. Common genetic factors were found through phenome-wide association studies between IgAN, IBD and bacterial infections. This leads to the hypothesis of a significant association between the gut microbiota's impact on immune system regulation and IgAN. Our previous study specifically focuses on the genetic aspects of the host gut microbiota 80. Out of 136 identified variations associated with gut microbiota, 9 were found to be linked to IgAN. Single nucleotide polymorphisms (SNPs) in genes *LYZL1, SIPA1L3, TTLL2, PLTP,* and *AL365503.1* were correlated with clinical parameters of IgAN. A SNP in *AL392086.3* was associated with poor prognosis. Specific SNPs in *LYZL1* were inversely correlated with the abundance of *Bacteroides*, while SNPs in *SIPA1L3* and *AL392086.3* were negatively associated with the abundance of *Proteobacteria*. SNPs in *TTLL2* were negatively linked to the abundance of *Anaerostipes*, whereas *PLTP* SNPs showed a positive correlation with *Veillonellaceae* abundance. Conversely, SNPs in *AL365503.1* and *RAD21-AS1* were positively related to the abundance of *Corynebacterium*. By involving two confirmation cohorts, we observed a decreased tendency for *Dialister* and an increased tendency for *Erysipelotrichaceae* in IgAN. The reduced abundance of *Dialister* consistently correlated with elevated serum levels of Gd-IgA1. These findings offer initial support for the notion that host genetics influence the gut microbiota in IgAN, suggesting a novel avenue for future research on pathogenesis.

By MR studies, it identified a likely causal relationship between gut microbiota-particularly specific bacterial taxa-and IgAN. Both Class *Actinobacteria* and Genus *Actinobacteria* are considered pathogenic factors in IgAN, while Genus *Enterorhabdus*, Family *Prevotellaceae*, and Family *Peptococcaceae* show protective effects against IgAN, with no indication of reverse causality 116, 117. This suggests that gut microbiota dysbiosis may be a significant factor in triggering or exacerbating the development and progression of IgAN. However, most of the national biobanks currently lack records of ICD codes for IgAN, or due to its low prevalence of IgAN within those biobanks, only few GWAS loci can be identified and validated in these databases, raising concerns about statistical power and result reliability from MR.

The genetics of the gut microbiome is still a field in its infancy, with only a few genetic loci have been consistently confirmed across multiple studies. However, we posit that discovering further host genetic factors affecting the gut microbiome, even those with minor impacts, will offer crucial understandings into intricate host-microbiome connections and could guide the development of therapies and individualized treatments. Future advancement in understanding the complex interactions by application of systems genetics (multi-omic) methodologies to both the human genome and the gut microbiome is necessary.

### 5.2. Gut microbiota: epigenetics effects

Epigenetics acts as a bridge between genotype and phenotype. Numerous studies have identified changes in DNA methylation, histone modifications, and non-coding RNAs that are closely linked to abnormal glycosylation of IgA1 and the production of Gd-IgA1 in IgAN. For instance, TRDMT1-driven 5mC RNA modification in B cells disrupts activation-induced cytidine deaminase activity and IgA class switch recombination (CSR), resulting in an exacerbated IgAN phenotype 118. Additionally, miR-374b, a miRNA targeting phosphatase and COSMC, promotes B-cell proliferation and aberrant IgA1 glycosylation when overexpressed 119. Unlike genetic mutations, epigenetic alterations are reversible and responsive to environmental factors. Sallustio *et al.* 120 suggested that elevated IL-6 levels in IgAN patients were induced by an epigenetic mechanism modulated by viral and bacterial RNA, which impacted the VTRNA2-1/PKR/CREB/IL-6 pathway.

The intricate interplay between epigenetics and the gut microbiota establishes a dynamic system, each highly responsive to environmental and dietary influences. The metabolites produced by gut microbiota act as cofactor and substrate for various enzyme reactions 121. Bacterial metabolites, such as SCFAs, have been shown to affect epigenetic markers like DNA methylation and histone acetylation directly 122. Epigenetic modifications, particularly miRNAs, can regulate the expression of genes that maintain intestinal barrier function, thereby influencing the types of bacteria that colonize the gut and impacting immune responses 123. The expression of miR-21-5p in intestinal epithelial cells may lead to changes in intestinal permeability 124. Casado-Bedmar *et al.* 125 identified that, in addition to impairing intestinal barrier function, the luminal increase of let-7b and miR-21 promotes the secretion of proinflammatory cytokines (TNF, IL-6, and IL-1β) by macrophages, enhances myeloperoxidase and antimicrobial peptide production, and ultimately contributes to intestinal dysbiosis by using an *in vitro* microbiota modeling system. Interestingly, miR let-7b, miR-21, and miRNA-21-5p have been shown to be involved in the production of IgA1 O-glycosylation in IgAN 126, 127. Furthermore, miRNAs seem to act as mediators between IgA CSR and the gut microbiota. Research by Casali *et al.* 128 has shown that in miR-146a-deficient mice, there are elevated IgA levels, an increased frequency of IgA^+^ B cells across various tissues, and notable IgA deposition in the kidneys. The loss of miR-146a enhances the recruitment of *Smad2*, *Smad3*, and *Smad4* to the *Igα* locus *Iα* promoter, a key step in initiating germline *Iα-Cα* transcription and CSR to IgA. Additionally, miR-146a-deficient chimeric mice exhibit significant alterations in gut microbiota composition, with marked increases in *Akkermansia*. Although studies specifically exploring the interaction between gut microbiota and epigenetics in the context of IgAN remain limited, current mechanistic insights strongly suggest that this interaction could be integral to IgAN development and progression. Investigating the gut-kidney axis through the examination of RNA methylation's impact on mucosal immunity in GALT, along with its interplay with the microbiome, may offer enhanced understanding of disease onset and advancement.

### 5.3. Gut microbiota: dysregulation of glycosylation by bacteria

The intestinal barrier, a mucus, epithelial, and immune layer composite, is integral to gut integrity. Its mucus component, rich in O-glycosylated mucins, segregates epithelial cells from luminal contents, including bacteria and antigens 129. Mucus contains a large amount of O-glycosylation, which makes up more than 80% of the mass of a mucin. O-glycan consists mainly of N-acetyl-galactosamine, N-acetyl-glucosamine, fucose, galactose, mannose and sialic acid, are all essential for barrier function 130.

Mucin2 (MUC2) is the main component of the intestinal mucus. Gut microbiota and metabolites influence the intestinal mucus barrier by modulating MUC2 synthesis, secretion, glycosylation, and other post-translational modifications 129. Within the luminal mucus layer, mainly constituted of elongated MUC2, commensal bacteria flourish by adhering to and metabolizing MUC2 glycans, with the assistance of glycan-degrading enzymes under normal physiological conditions. The expression of NHE3 is regulated by SCFAs, thereby facilitating the development of a dense inner mucus layer that lies adjacent to epithelial cells 131. Additionally, activating AHR by indole derivatives stimulates tight junction protein expression and mucin production 132. Studies conducted earlier have proposed that the group of mucin-degrading bacteria is mainly composed of *A. muciniphila*, *Bacteroides thetaiotaomicron*, *B. fragilis*, *Bifidobacterium bifidum*, *R. gnavus*, and *R. torques* 133. This list is likely to expand, as 23 representative gut microbes have been shown to utilize porcine intestinal mucin as their sole carbon source for growth 134. The proliferation of mucus-degrading bacteria can exacerbate the degradation of MUC2, thereby triggering intestinal inflammation 135. *R. gnavus,* known for its abundance of genes encoding carbohydrate-active enzymes, has been observed to modify mucin O-glycosylation patterns in individuals with IBD, a discovery that could have implications for IgAN 136.

In IgAN, alternations in the intestinal barrier, specifically increased permeability, have been recorded 137. The glycosylation pattern of IgA1 in IgAN, mainly core-1, might be influenced by variations in enzymes such as β-galactosyltransferase and cosmc 138. Analysis of serum IgA tryptic glycopeptides has identified various N-glycosylation structural characteristics, including differences in galactosylation, sialylation, bisection, fucosylation, and N-glycan complexity, which are associated with IgAN and renal function 139. These findings highlight a potential role of mucin dysregulation in IgAN pathogenesis, where aberrant glycosylation and increased mucosal permeability may promote pathogenic IgA production. Further investigation into the mechanisms by which these bacteria alter mucin structure and function could provide valuable insights into their role in the development of IgAN.

### 5.4. Gut microbiota: molecular mimicry and microbial metabolites

#### Molecular mimicry

Certain bacterial antigens may possess amino acid sequences or molecular structures that resemble self-antigens, such as major histocompatibility complex molecules. This similarity can lead to the over-activation of auto-reactive immune cells, which may mistakenly target and attack human tissues, contributing to autoimmune responses 140. This mechanism, known as “molecular mimicry”, is thought to play a role in various autoimmune diseases, including Guillain-Barre syndrome 141 and systemic lupus erythematosus 142. Some human leukocyte antigen polymorphisms are recognized as risk factors for IgAN and may predispose individuals to antibody responses against specific environmental pathogens or contribute to a loss of immune tolerance 143. Several environmental microbes, including those with polysaccharides displaying the GalNAc motif on their cell surface, can prime B cells to produce IgA and IgG antibodies targeting these structures. Such antibodies could cross-react with the hinge region of Gd-IgA1. Infection by EB virus, respiratory syncytial virus, herpes simplex virus, and streptococci may induce the production of such antibodies 144. Nihei *et al.* 145 showed that certain oral bacteria can elicit immune responses that produce IgA capable of cross-reacting with mesangial cells, thereby initiating the development of IgAN. Moreover, in the grouped ddY spontaneous IgAN mouse model, IgA^+^ plasmablasts accumulate in the kidneys, where they produce IgA targeting mesangial antigens, including βII-spectrin and CBX3 146, 147. This finding supports the idea that local IgA production against mesangial antigens plays a direct role in kidney damage in IgAN. It remains unclear whether specific antigens from the gut microbiota cross-react with IgAN, but this hypothesis is gaining attention and may be of interest in formulating a vaccine to prevent the onset of diseases.

#### Microbial metabolites

Metabolites are pivotal in the regulation of inflammation in both the intestinal and parenteral settings through their influence on leukocyte recruitment and chemokine function. SCFAs alter cell recruitment by modulating the expression of adhesion molecules in neutrophils and endothelial cells. Particularly, propionate and butyrate have been observed to suppress pro-inflammatory agents like TNF-α, IL-6, and nitric oxide. Conversely, butyrate boosts IL-10 expression, facilitating immune tolerance in lymphocytes 148. The presence of low concentrations of butyrate promotes the release of MUC2 from intestinal epithelial cells, enhancing the barrier function and the ability to respond to pathogens and commensal microorganisms. Conversely, high concentrations of butyrate have been shown to impair the barrier function 149. SCFAs also fuel B cells to augment IgA production and activate dendritic cells through SCFA receptor engagement and histone deacetylase inhibition, facilitating IgA CSR 150. There is a reduction in fecal levels of SCFAs from patients with IgAN, including acetic, propionic, butyric, isobutyric, and caproic acids, which is associated with a decline in SCFA-producing bacteria like *Alistipes* 105. The implications of SCFAs in IgAN may encompass heightened intestinal permeability, diminished expression of antimicrobial peptides, inflammatory activation, and increased susceptibility to pathogen infections 151.

Tryptophan, an essential amino acid sourced from dietary proteins, undergoes metabolism via host (kynurenine and serotonin) and microbial (indole) pathways 152. In IgAN, elevated levels of 5-hydroxytryptophan and kynurenine, alongside reduced indole metabolites such as indole-3-acetic acid and 3-indolepropionic acid, have been reported 106. Lower levels of 3-indolepropionic acid in the intestine impair the integrity of the intestinal barrier, causing elevated permeability and the activation of inflammatory processes 153. Moreover, decreased intestinal 3-indolepropionic acid levels have been associated with increased intestinal SIgA and IgG in *Clostridium* sporogenes-deficient mice 154.

### 5.5. Gut microbiota: B cell activation

Intestinal B cell activation and differentiation rely heavily on the gut microbiota. In return, B cells help regulate the gut microbiota and maintain intestinal homeostasis through the production of immunoglobulins. The role of IgA in shaping microbiota was initially identified in mice deficient in activation-induced cytidine deaminase (AID), an enzyme essential for antibody isotype switching. AID-deficient mice exhibited hyperplasia of the intestinal lymphoid follicles and a 100-fold increase in anaerobic commensal bacteria within the intestine 155. Bacterial flow cytometry and 16S rRNA gene sequencing have identified a diverse set of IgA-coated microbiota, including *Actinomyces*, *Bifidobacterium*, *Erysipelotrichaceae*, *Dorea*, *Ruminococcus*, *Akkermansia*, *Streptococcus*, *Escherichia-Shigella*, *Clostridium*, *Bacteroides*, *Blautia* and *Roseburia* 156. Studies suggest that *Bacteroides* species elicit a stronger IgA response in murine Peyer's patches compared to *Lactobacillus*, possibly through the upregulation of AID in B cells 157. *Bacteroides ovatus*, in particular, has been shown to stimulate significant mucosal IgA production through a T cell-dependent B cells activation pathway 158.

Extracellular vesicles derived from high-protein-fed microbiota activate epithelial TLR4 and promote the expression of BAFF and a proliferation-inducing ligand (APRIL) 159. Morphine-induced gut microbial dysbiosis triggers TLR-dependent IgA targeting gram-positive bacteria and induces upregulation of CD11b and TLR2 on a specific subset of IgA^+^ B cells 160. This suggested that B cells were regulated by dietary metabolites. The MyD88 signaling pathway is downstream of TLR receptors. MyD88-mediated signaling was required for the development of intestinal IgA^+^ B cells. Loss of Disruption of MyD88 signaling diminished targeting of the gut microbiota by high-affinity IgA leading to a breakdown in the regulation of bacterial growth and community homeostasis 161.

Additionally, dysfunction of the epithelial barrier can lead to abnormal B cell immune responses. A recent study by Kinashi *et al.* 162 provides evidence that Ap1m2 deficiency induces intestinal epithelial barrier dysfunction and resulting dysbiosis, which spontaneously lead to IgAN-like features in the mouse kidney. Moreover, Ap1m2 deficiency resulted in a marked increase in IgA^+^ B cells within the gut lamina propria, accompanied by elevated IgA levels in the supernatant of *ex vivo* intestinal cultures. This enhanced mucosal IgA response in Ap1m2 deficiency mice is likely driven by intestinal dysbiosis, characterized by an overabundance of *Candidatus Arthromitus*. *Candidatus Arthromitus*, previously identified as a segmented filamentous bacterium, is a powerful stimulator of the intestinal immune system, notably enhancing Th17 and IgA responses 163. Subsequently, the depletion of gut microbiota through antibiotic treatment reduced IgA deposition in the kidneys of Ap1m2 deficiency mice.

## 6. Translational research in IgAN

### 6.1. Biomarkers

#### 6.1.1. Traditional biomarkers in IgAN

Over recent decades, the diagnostic and prognostic landscape of IgAN has relied heavily on non-specific biomarkers. The cornerstone of IgAN diagnosis remains in kidney biopsy, a method with inherent limitations due to its invasiveness and potential complications. In predicting IgAN progression, clinicians have traditionally used a combination of non-specific markers such as proteinuria, blood pressure, and eGFR, supplemented by IgAN-specific findings from kidney biopsy. Among these, the Oxford MESTC histologic score stands out. This score encapsulates four key pathological features: mesangial, endocapillary hypercellularity, segmental sclerosis, and interstitial fibrosis/tubular atrophy. Each component contributes to a comprehensive understanding of the disease's severity and progression risk 164. Its significance is underpinned by 21 validation studies involving nearly 7,000 patients across various continents, establishing its robustness in clinical practice 165. The development of the International IgAN Prediction Tool marks a significant advancement in prognostic strategies 166. This tool amalgamates globally available, clinically embedded biomarkers validated for prognostic efficacy. Its recent validation in a cohort of 1,275 patients further underscores its potential utility in clinical settings 167. Nevertheless, neither pathological evaluations nor International IgAN Prediction Tool can guide treatment strategies or facilitate real-time disease surveillance.

#### 6.1.2. Intestinal barrier biomarkers in IgAN

Recent advancements in the study of IgAN have highlighted the importance of intestinal barrier biomarkers. Research conducted by our team has revealed elevated levels of serum zonulin in IgAN patients. This elevation points to zonulin's crucial role as a regulator of epithelial and endothelial barrier functions, thereby emphasizing its potential as a biomarker in this disease context 168. Zhou *et al.* 169 explored the characteristics of the intestinal barrier in rats with IgAN. Their study identified a strong correlation between the degradation of the intestinal barrier and reduced expression of the tight junction proteins zonula occludens-1 and occludin, plus intestinal microbiota dysbiosis in IgAN rats. In the IgAN mice model, rhein was observed to enhance the expression of zonula occludens-1 and occludin, which is crucial for repairing damaged tight junctions and restoring the intestinal barrier 170.

#### 6.1.3. Microbiomic biomarkers in IgAN

The preceding sections have outlined differential findings regarding the gut microbiome in individuals with IgAN compared to healthy individuals and across varying disease stages. These observations propose that changes in specific microbial taxa, the overall structure of the microbial community, diminished bacterial diversity, and the stability of the microbial community may hold potential as biomarkers for IgAN. A recent study has documented that a striking expansion of the taxonomic chain *Proteobacteria*-*Gammaproteobacteria*-*Enterobacteriales*-*Enterobacteriaceae*-*Escherichia-Shigella* was observed in patients with IgAN who were treatment-naive, which was reversed only in patients who achieved clinical remission after six months of immunosuppressive therapy. The study suggests *Escherichia-Shigella* test in patients with IgAN may be utilized as a tool for both differential diagnosis and monitoring the effectiveness of immunosuppressive therapy 92.

### 6.2 Therapy targeting microbiota

**Figure 4:**
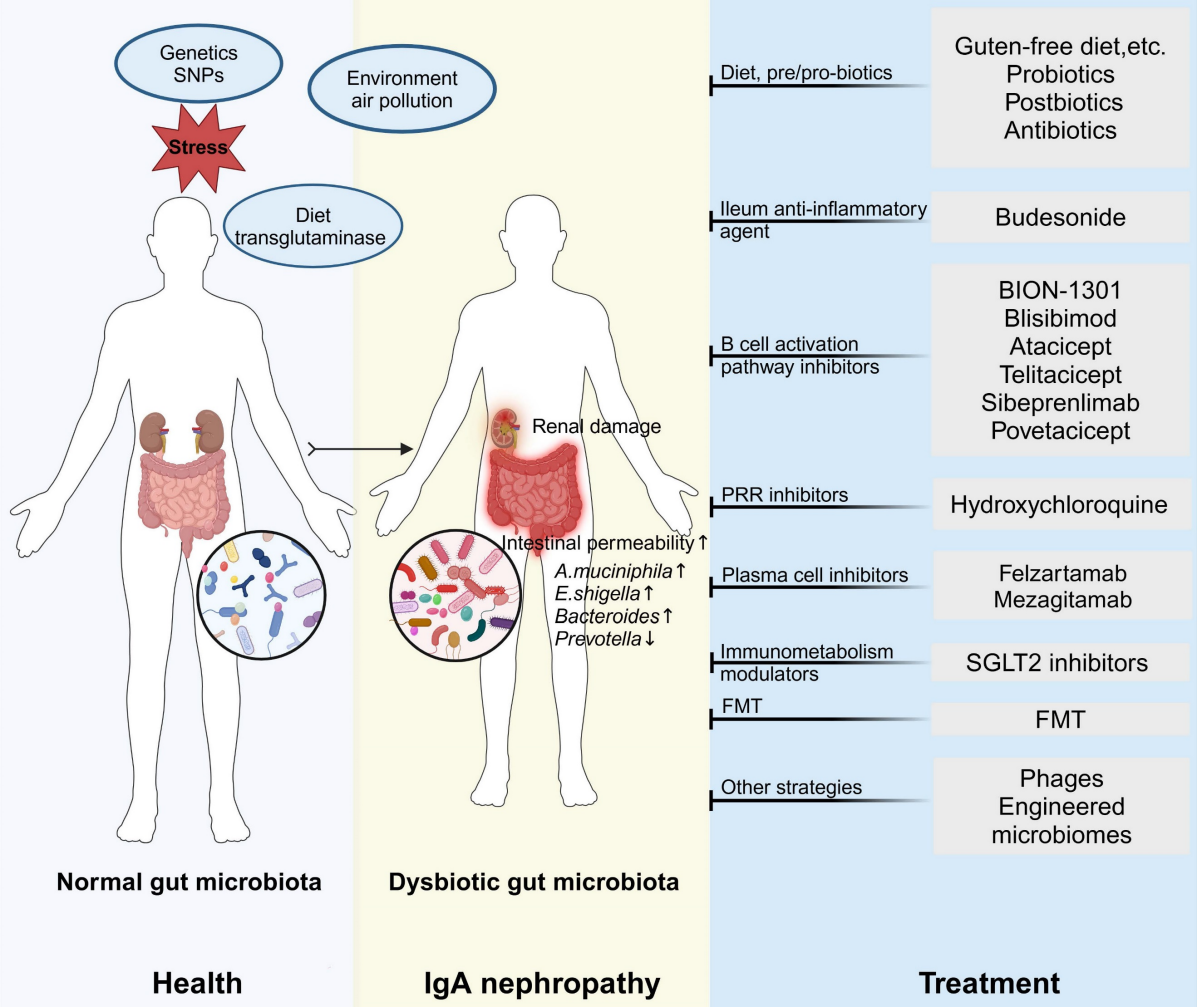
New prospective treatments targeting the intestinal mucosal immune system in IgA nephropathy. Abbreviations: FMT: fecal microbiota transplantation; PRRs: pattern recognition receptors; SGLT2: sodium-glucose co-transporter 2; SNPs: single nucleotide polymorphisms. Created with BioRender.com.

The gut microbiome, dynamic and diverse, is heavily subject to external modulation. The presence, function, and interaction of bacteria with the host, diet, and various gut components can significantly affect the development of infectious and chronic diseases. This underscores the potential of the gut microbiota as a novel therapeutic target for IgAN. Emerging evidence suggests the efficacy of microbiota-focused interventions in ameliorating IgAN **(Figure 4)**.

#### 6.2.1 Dietary interventions, antibiotics, prebiotics and probiotics

The impact of diet on the gastrointestinal tract, in terms of regulating gut microbiota composition and functionality, as well as the influence of inadequate nutrition on the pathogenesis and progression of several disorders, has been extensively documented. A MR study has confirmed that alcohol intake frequency is associated with an increased risk of IgAN, whereas the intake of cheese, cereal, and sushi is associated with a decreased risk of IgAN 171. High-fat, high-sugar, high-salt, and high-animal protein diets can contribute to the proliferation of pathogenic bacteria in the gut, leading to gut dysbiosis, inflammation, and compromised intestinal barrier integrity. On the contrary, a diet abundant in vegetables and fibers, supplemented with probiotics and vitamin D, leads to the restoration of gut microbiota and an elevation in anti-inflammatory factors associated with the microbiome, such as SCFAs. Clinical observations revealed a decline in IgA antigliadin antibodies and proteinuria in IgAN patients upon adoption of a gluten-free diet 172. Of interest, the protective effects of the Mediterranean diet against a range of conditions, including chronic inflammatory disorders such as IgAN, have been documented. These benefits are attributed to its ability to suppress pro-inflammatory factors (IL-1, IL-6) and reduce oxidative stress 173. In light of the significant impact of diet on the composition of gut microbiota, crucial for preserving normal immune responses and kidney health, lowering risks, modulating symptoms, and ameliorating pathophysiological factors linked to IgAN, the integration of dietary adjustments with pharmacological interventions could serve as a viable strategy to rebalance gut microbiota dysbiosis and enhance symptomatic relief.

Antibiotics may have the potential to be utilized for modulating the gut microbiota as a practical therapeutic intervention in IgAN. Previous research has illustrated that antibiotic treatment significantly reduced hIgA1 mesangial deposition, glomerular inflammation, and the progression of proteinuria in the α1KI-CD89Tg mouse model 174.

Probiotics exhibit antimicrobial and anti-inflammatory properties, and they also reduce intestinal permeability, aiding in the maintenance of intestinal microbiota balance and alleviation of gastrointestinal issues. The use of probiotics is recommended for addressing intestinal disorders such as IBD, celiac disease, as well as various cardiovascular diseases, and obesity 175. In individuals with IgAN, shifts in the gut microbiome have been documented, characterized by elevated levels of *A. muciniphila* and *Streptococcus*, and diminished populations of butyrate-producing bacteria 30. It seems reasonable that probiotics such as *Lactobacillus plantarum*, and *Bifidobacterium pseudocatenulatum* may function as adjuncts in countering the onset and progression of IgAN, due to their anti-inflammatory and antioxidant properties. In concordance, the administration of *Bifidobacterium* as a supplement offers promise in alleviating the clinicopathological manifestations of IgAN by impeding the NLRP3 signaling pathway and mitigating gut dysbiosis, characterized by an augmentation of beneficial bacteria and a reduction in potentially pathogenic bacteria, as demonstrated in an IgAN mouse model 101.

#### 6.2.2 Budesonide

Despite the well-established "four-hit hypothesis," numerous mechanisms contributing to disease pathogenesis remain inadequately described, including B-cell priming triggered by various antigens within the intestinal microbiota. In the human body, several sites harbor organized lymphoepithelial tissue, including the tonsils. Nonetheless, the most crucial locations for IgAN are the GALT and Peyer's patches, where B cells in the ileum's mucosal layer produce Gd-IgA1 in response to dietary (e.g., gluten) or microbial antigens 176. The correlation demonstrates the potential efficacy of budesonide in addressing intestinal immunity and localized inflammation in the context of IgAN treatment. A budesonide formulation designed to specifically deliver the drug within the intestine to immune cells producing IgA (Nefecon), was first utilized as a novel therapeutic intervention for IgAN ten years ago 177. A compilation of recent reviews suggests that previous research backs the prescription of budesonide for IgAN treatment, showing a decrease in proteinuria and the stability of renal function 178. The results of the global phase 3 clinical trial (ClinicalTrials.gov identifier: NCT03643965) demonstrated a statistically significant treatment benefit with Nefecon versus placebo by the time-weighted average of eGFR over two years 179. Notably, a 9-month treatment period with Nefecon provided a clinically relevant reduction in eGFR decline and a durable decrease in proteinuria versus placebo, supporting a disease-modifying effect in patients with IgAN. Nefecon has been proven to reduce pathogenic forms of IgA and IgA immune complexes.

#### 6.2.3. B/Plasma cell depletion/modulation

Recent research has provided compelling insights into the role of plasma cells in IgAN. A noteworthy study confirmed that patients with IgAN exhibit elevated circulating surface Gd-IgA1^+^ B cells expressing the chemokine receptors CCR10 and CCR9. These receptors are closely associated with the upper respiratory tract and gut. Furthermore, it was observed that the Gd-IgA1^+^ cell population in peripheral blood is enriched with plasma cells 180. These analyses indicate that B cell subpopulations and serum Gd-IgA1 could be explored as novel biomarkers for treating IgAN.

Therapeutic strategies for targeting Gd-IgA1-producing B cells may be summarized in two points: (1) Direct removal of B cells, including debulking of MALT-Tonsillectomy, GALT targeting-CD38/40 monoclonal antibody (2) Modulation of the B cell programming involved in the abnormal IgA production, including Corticosteroid, Proteasome inhibitor, TLR antagonism, APRIL/BAFF antagonism.

Some ongoing trials are testing emerging drugs that can interfere with plasma cells. For instance, CD38-directed therapies that target and deplete plasma cells including felzartamab (ClinicalTrials.gov identifier: NCT05065970) and mezagitamab (ClinicalTrials.gov identifier: NCT05174221), inhibits of BAFF and APRIL, including BION-1301 (ClinicalTrials.gov identifier: NCT03945318), Blisibimod (ClinicalTrials.gov identifier: NCT02062684), Atacicept (ClinicalTrials.gov identifier: NCT02808429), Sibeprenlimab (ClinicalTrials.gov identifier: NCT05248646/NCT05248659), Telitacicept (ClinicalTrials.gov identifier: NCT04905212), and Povetacicept (ClinicalTrials.gov identifier: NCT06564142) have been demonstrated to have significant therapeutic effects in IgAN. Furthermore, our previous randomized controlled trial has provided evidence of the therapeutic potential of oral Hydroxychloroquine. Hydroxychloroquine, an inhibitor of mucosal and intrarenal TLRs administered for six months, has shown remarkable effectiveness in significantly reducing proteinuria levels in patients with IgAN 181. It is still awaiting international randomized controlled trials and long-term follow-up data in determining its efficacy and safety in improving kidney outcomes.

#### 6.2.4. Sodium-Glucose Transporter 2 Inhibition

Given that IgAN is a common cause of glomerular disease and CKD, large numbers of patients with IgAN were included in the DAPA-CKD and EMPA-KIDNEY trials of SGLT2 inhibitors in non-diabetic CKD. In these trials, SGLT2 inhibitor treatment produced substantial benefits for IgAN patients, slowing kidney disease progression and improving survival outcomes 182, 183. SGLT2 inhibitors are thought to exert nephroprotective effects through mechanisms such as tubuloglomerular feedback-induced vasoconstriction of afferent arterioles and increased proximal tubular pressure, both of which contribute to lowering glomerular capillary pressure and reducing renal oxygen consumption 184. A recent study suggests that SGLT2 inhibitors may regulate the gut microbiota, reducing the production of uremic toxins and thereby exerting nephroprotective effects 185. Another study has also confirmed that SGLT2 inhibitors, empagliflozin, mitigates DN by modulating the gut microbiota, leading to a reduction in LPS-producing bacteria and an increase in SCFA-producing bacteria 186. The renoprotective effect of SGLT2 inhibitors is beyond doubt, its association with gut microbes in IgAN treatment is worthy of detailed exploration.

#### 6.2.5. Fecal Microbiota Transplantation

In recent times, there has been an increasing focus on FMT as a viable and efficacious method to restore eubiosis in numerous illnesses. Initially sanctioned for addressing *Clostridium difficile* infection, FMT is now being explored for a range of gastrointestinal and non-gastrointestinal conditions 187. FMT offers a distinct advantage over probiotics, prebiotics, and synbiotics due to its capacity to confer enduring benefits following a solitary intervention, with the option of repetitive administrations based on individual assessments. Additionally, numerous studies have documented a therapeutic effectiveness coupled with minimal side effects. Notably, two case studies suggest that these individuals exhibited a notable decrease in 24-hour urine protein levels over a six-month period post-treatment, alongside a progressive augmentation in the diversity of their gut microbiota subsequent to FMT 188, 189. Another study indicates that after FMT, a decrease in the absolute count of serum B cells was observed. Notably, changes in the relative abundance of *Bacteroides uniformis* and *Bacteroides ovatus* showed a significant positive correlation with serum B cell count changes, while the abundance change of *Prevotella copri* was significantly negatively correlated with serum B cell counts 190. Beyond transplanting a raw fecal lysate, specific fecal components, such as certain miRNAs that regulate microbiota or particular bacterial strains, may also be utilized as targeted treatment 191.

## 7. Conclusions and future perspectives

In this comprehensive review, we have summarized the observed alterations in the gut microbiota and associated metabolic pathways in the context of IgAN. We have also delved briefly into the underlying mechanisms driving these alterations and extensively discussed the potential of the gut microbiome as a groundbreaking therapeutic target for the treatment of IgAN. Over recent years, a wealth of research has elucidated the profound impact of gut microbiota and its metabolites on the pathogenesis of IgAN. As we navigate the future of IgAN research, it becomes increasingly evident that microbiota-based diagnostics and therapeutics hold tremendous promise.

In moving the field forward, many vital challenges need to be addressed, and some recommendations provided might be helpful (1) Investigating the causal link between the gut microbiome and IgAN pathogenesis is crucial. Establishing strong evidence of causality will deepen our comprehension and facilitate the development of precise interventions. (2) Understanding the complex interplay between the gut and kidneys requires a detailed exploration of the underlying mechanisms. It is crucial to pinpoint specific microbial strains that significantly maintain mucosal integrity and produce Gd-IgA1. (3) Understanding the complex interplay between the gut and kidneys necessitates a detailed exploration of the underlying mechanisms. To unravel this intricate relationship, it is essential to pinpoint specific microbial strains responsible for maintaining mucosal integrity and producing Gd-IgA1. (4) The advancement of microbiota-based treatment strategies in IgAN relies heavily on developing novel methodological tools. Targeted metabolomics, engineered microbial strains, and bacteriophages are emerging as promising avenues in microbiome research, with the capacity to transform the management of IgAN. (5) Personalized microbiota modulation therapy by investigating a tailored approach in IgAN patients.

The dysregulation of gut microbiota is implicated in the pathogenesis of IgAN, potentially triggering abnormal IgA production, renal inflammation, and functional impairment. Modulating gut microbiota balance presents a novel therapeutic avenue for IgAN, involving strategies like probiotics, FMT, and dietary modifications. Future research will uncover the intricate link between gut microbiota and IgAN, fostering the development of personalized treatment modalities. Emphasis in research and treatment may shift towards multi-organ interventions, especially in systemic therapies targeting gut microbiota, inflammation, and hemodynamics, potentially leading to substantial improvements in long-term patient prognosis.

## Figures and Tables

**Table 1:** Altered gut microbiota compositions, and fecal and/or serum metabolite in IgA nephropathy.

Study	Country	IgAN /HCs (N/n)	Methods	Key findings					
				gut microbiota		fecal metabolites		serum metabolites	
				Increased	Decreased	Increased	Decreased	Increased	Decreased
Sui, 2012 107	China	35 (23IgAN-A,12 IgAN-B)/23	Proton nuclear magnetic resonance spectroscopy	Not assessed		Not assessed		phenylalanine, myo-Inositol, lactate, L6 lipids ( = CH-CH2-CH = O), L5 lipids (-CH2-C = O), L3 lipids (-CH2-CH2-C = O)	β-glucose, α-glucose, valine, tyrosine, phosphocholine, lysine, isoleucine, glycine, glutamine, glutamate, alanine, acetate, 3-hydroxybutyrate
De Angelis, 2014 96	Italy	32 (16 NP, 16 P)/16	16S (V1-V3); GC-MS/SPME	Firmicutes, Ruminococcaceae, Lachnospiraceae, Eubacteriaceae, Streptococcaeae, Sutterellaceae, Escherichia sp.	Bifidobacterium, Clostridium, Enterococcus, Lactobacillus, Leuconostoc, Bacteroidetes, Prevotellaceae.	FAA (Glu, Ala, Asp, Val, Leu, Pro), ethyl alcohol, 2,6-octadien-1-ol 3,7 dimethyl- (Z), 1-octanol, 4-methyl-phenol, phenol 4- (1,1,3,3- tetramethylbutyl)	aldehydes, tridecanal, ketons	Not assessed	
Dong, 2020 89	China	44/33	16S (V3- V4)	Escherichia-Shigella	Roseburia, Clostridium, Fusobacterium	Not assessed		Not assessed	
Hu, 2020 90	China	17/16	16S (V3-V4)	Escherichia-Shigella, Eggerthella	Coprococcus, Barnesiella, Prevotellaceae	Not assessed		Not assessed	
Zhong, 2020 82	China	52/25	16S (V3- V4)	Fusobacteria, Bacteroides, Escherichia-Shigella	Firmicutes, Actinobacteria, Blautia, Prevotella 9, Bifidobacterium	Not assessed		Not assessed	
Chai, 2021 105	China	29/29	16S (V3-V4); GC/MS	Actinobacteria, Eggerthella, Alloprevotella, Enterococcaceae, Streptococcus, Blautia	Prevotellaceae, Alistipes, Lachnospira	NS	acetic acid, propionic acid, butyric acid, isobutyric acid, caproic acid	Not assessed	
He, 2021 80	China	87/24; 27/19	16S (V3- V4)	Bacteroides	Dialister, Prevotella	Not assessed		Not assessed	
Sugurmar, 2021 192	Malaysia	36/12	16S (V3-V4)	Fusobacteria, Epsilonproteobacteria	Euryarchaoeota, Methanobacteria	Not assessed		Not assessed	
Shah, 2021 94	France	20/20	16S (V3-V4)	Bacteroides, Escherichia-Shigella	Prevotella9, Ruminococcaceae	Not assessed		Not assessed	
Wu, 2021 102	China	15/30	16S (V3-V4); LC-MS/MS	Blautia, Streptococcus, Enterococcus	Faecalibacterium, Bacteroides, Prevotella9, Dialister	bilirubin, trimethoprim, phenylalanine, phosphatidylethanolamine (PE lyso 17:0)	stearamide, cis-9,10-epoxystearic acid etc.	Not assessed	
Dong, 2022 91	China	117/150	16S (V3-V4); LC-MS	Proteobacteria, Actinobacteriota, Escherichia-Shigella, Streptococcus, Bifidobacterium, Dorea, Roseburia, Collinsella	Anaerostipes, Parasutterella, Fusicatenibacte, Blautia, Lachnospira, Bacteroides	Not assessed		myo-Inositol, (1H-Indol-3-yl)-N-methylmethanamine, catechol, pimelic acid, oxaloglutarate, tryptophan etc.	folic acid, octadecanamide, l-tyrosine, beta-Alanine, Cholesterol, etc.
Tang, 2022 95	China	35/20	16S (V3-V4)	Escherichia-Shigella, Bacteroides	Actinobacteria, Bifidobacterium, Blautia	Not assessed		Not assessed	
Zhao, 2022 92	China	127/127	16S (V3-V4)	Proteobacteria, Escherichia-Shigella, Pseudomonas, Erysipelatoclostridim	Lachnospira, Lachnospiraceae, Fusicatenibacter, Agathobacter, Romboutsia	Not assessed		Not assessed	
Wu, 2022 106	China	15/30	16S (V3-V4); LC-MS/MS	NS	Bacteroidetes	oligopeptides, polypeptides, phenylalanine, tryptophan, tyrosine, leukotriene B4, leukotriene D4.	cycloleucin, 3-indolepropionic acid, palmitoleic acid, oleic acid, 9-OxoODE	citrulline, arginine, ornithine, indoxyl-sulfate, phenylacetylglutamine, indole, 3-hydroxyanthranilic acid, xanthurenic acid, kynurenine	creatinine, guanidinosuccinic acid, putrescine, 3-indolepropionic acid, indoleacrylic acid, anthranilic acid
Tan, 2022 101	China	35/25	16S (V3-V4)	Bacteroides	Bifidobacterium, Prevotella 9	Not assessed		Not assessed	
Liang, 2022 81	China	20/20	Metagenomic sequencing	Bacteroides, Flavonifractor, Bacteroides fragilis, Flavonifractor plautii, Ruminococcus gnavus, bacteroides vulgatus	Alistipes, Prevotella, Faecalibacterium, Ruminococcus, Alistipes putredinis, Faecalibacterium prausnitzii, Prevotella copri	Not assessed		Not assessed	
Tang, 2023 100	China	25/20	16S (V3-V4)	Proteobacteria, Fusobacteria, Bacteroides, Faecalibacterium, Ruminococcus, Escherichia-Shigella.	Bifidobacterium, Blautia, Roseburia, Coprococcus	Not assessed		Not assessed	
Bao, 2023 93	China	19/15	16S (V3-V4)	Escherichia-Shigella, Bifidobacterium, Dorea	Bacteroidetes, Lachnospira, Coprococcus, Sutterella.	Not assessed		Not assessed	
Cai, 2023 193	China	260/174	16S (V3-V4)	NS	Butyricicoccus, Coprococcus, Ruminococcus	Not assessed		Not assessed	
Jeon, 2023 108	Korea	20 (10NP, 10 P)/10	Proton nuclear magnetic resonance spectral	Not assessed	Not assessed	Acetone, Glycerol, Glycine, Threonine, Valine, Formate, Betaine, N,N-Dimethylglycine.	NS	Not assessed	
Zhu, 2024 99	China	48/31	16S (V3-V4)	Escherichia-Shigella, Clostridium	NS	Not assessed		Not assessed	
Gao, 2024 98	China	77/69	16S (V3-V4)	Escherichia-Shigella, Bacteroides, Alistipes	Faecalibacterim, Prevotella	Not assessed		Not assessed	
Gleeson, 2024 30	France	33/65	16S (V3-V4)	Akkermansia muciniphila, Ruminococcus, Blautia	Prevotella, Parabacteroides	Not assessed		Not assessed	
Yuan, 2024 97	China	61/68	16S (V3-V4)	Bacteroides, Escherichia-Shigella, Parabacteroides	Parasutterella, Dialister, Faecalibacterium, Subdoligranulum	Not assessed		Not assessed	

Abbreviations: GC-MS/SPME: Gas-chromatography mass spectrometry-solid-phase microextraction; HC: healthy controls; IgAN-A: diseases of grades I-III based on renal biopsies stained; IgAN-B: diseases of grades IV-V based on biopsies stained; LC-MS/MS: liquid chromatography-tandem mass spectrometry; NP: non-progressor; P: progressor. NS: no significance.

**Table 2:** Characterization of the gut microbiota in IgAN animal models

Study	Country	Model	Key words	Key findings
McCarthy, 2011 109	Canada	BAFF-Tg mice	Commensal flora, IgA-associated nephropathy	Serum IgA from ASF-colonized BAFF-Tg mice bound specifically to Lactobacillus murinus isolated from these mice. After colonization of BAFF-Tg mice with ASF, the number of IgA^+^ B220^-^ B cells were highest in the BAFF-Tg lamina propria compartment.
Chemouny, 2019 110	France	α1KI-CD89Tg mice	Antibiotics, IgAN, Gut microbiome	Antibiotic treatment efficiently depleted the fecal microbiota, impaired GALT architecture and impacted mouse IgA production. The antibiotic treatment markedly prevented hIgA1 mesangial deposition, glomerular inflammation and the development of proteinuria. Fecal bacterial load strongly correlated with critical clinical and pathophysiological features of IgAN such as proteinuria and hIgA1-mIgG complexes.
Fukunaga, 2019 114	Japan	Grouped ddY mice	Dietary lipid, Dietary protein, Gut microbiome	Abundance levels of Desulfovibrionaceae sp., Oscillospira, and Bacteroides were high in mice fed a diet containing 20% milk casein and 17% beef tallow. Faecalibaculum rodentium- and Allobaculum stercoricanis-like bacteria were highly abundant in the mice fed 40% whole-egg powder.
Fukunaga, 2020 115	Japan	Grouped ddY mice	Beef tallow, Casein, Egg yolk, Gut microbiome	L. murinus- and B. vulgatus-like bacteria were susceptible indigenous bacteria to egg yolks. Lachnospiraceae-like bacteria was susceptible indigenous bacteria to diet containing either 20% (w/w) milk casein and 17% beef tallow.
Di Leo V, 2021 112	France	α1KI-CD89Tg mice	Gut microbiome, Rifaximin	Rifaximin treatment decreased the urinary protein-to-creatinine ratio, serum levels of hIgA1-sCD89 and mIgG-hIgA1 complexes, hIgA1 glomerular deposition, and CD11b^+^ cell infiltration. Rifaximin treatment decreased significantly BAFF, and TNF mRNA expression.
Lauriero, 2021 111	France	α1KI-CD89Tg mice	FMT, IgAN, Gut microbiome	The microbiota from P-pts was able to induce an increase of serum BAFF and galactose deficient-IgA1 levels and a decrease of CD89 cell surface expression on blood CD11b^+^ cells which was associated with soluble CD89 and IgA1 mesangial deposits. The microbiota from HC-sbjs induced a decrease in albuminuria, increased CD11b^+^ cell surface CD89 expression and reduced expression of renal inflammatory chemokines.
Kano, 2021 194	Japan	Grouped ddY mice	Germ-free, IgAN, Aberrantly glycosylated IgA	The germ-free IgAN-onset ddY mice nasally immunized with CpG-oligonucleotide showed aggravation of kidney injury with mesangial IgA deposition, whereas those that received fecal transplants did not develop IgAN. The germ-free IgAN-onset ddY mice did not develop IgAN, while they showed aggravation of kidney injury with mesangial IgA deposition after transfer to the specific pathogen-free state.
Tan, 2022 101	China	W-IgAN mice	Gut dysbiosis, IgAN model	Both supplementation with probiotics mainly containing Bifidobacterium and their SCFA metabolites could attenuate the clinicopathological manifestations of IgAN by inhibiting the NLRP3/ASC/Caspase 1 signaling pathway.
Currie, 2022 195	Canada	BAFF-Tg, HC-Tg, B×hC-Tg mice	Cytokines, Immunoglobulins, Immunology	Colonization of B×hC-Tg mice with Neisseria resulted in elevated levels of systemic Neisseria-specific IgA. Neisseria-specific IgA-secreting cells were detected within the kidneys of these mice.
Xie, 2022 196	China	α1KI-Tg mice	IgA protease, Fc-fusion protein, IgAN	A recombinant fusion IgA protease derived from the commensal gut microbiota Clostridium ramosum was able to eliminate chronic IgA and associated complement C3 deposits in the glomeruli.
Gleeson, 2024 30	France	α1KI-CD89Tg mice	Akkermansia muciniphila, IgAN	Mice expressing human IgA1 and the human Fc α receptor I (α1KI-CD89tg) that underwent intestinal colonization by Akkermansia muciniphila developed an aggravated IgAN phenotype.
Zhu, 2024 99	China	C57BL/6J	FMT, IgAN	Mice colonized with gut microbiota from IgAN patients mimicked the IgAN phenotype with the activation of TLR4/MyD88/nuclear factor-κB pathway and B-cell stimulators in the intestine.

Abbreviations: ASF: altered Schaedler flora; BAFF: B cell activation factor of the TNF family; BAFF-Tg mice: BAFF overexpressing transgenic mice; B×hC-Tg: BAFF× hC-Tg progeny; FMT: fecal microbiota transplantation; GALT: gut-associated lymphoid tissue; HC-Tg: human CEACAM-1 transgenic mice; HC-sbjs: healthy controls; MyD88: Myeloid differentiation factor 88; NALT: nasal-associated lymphoid tissue; NP-pts: non-progressor; P-pts: progressor; TLR4: toll-like receptor 4; W-IgAN mice: with bovine serum albumin (BSA), tetrachloromethane, castor oil, and lipopolysaccharide (LPS) for 8 consecutive weeks. α1KI-CD89Tg mice: humanized mouse model of IgAN.

## Abbreviations

AHR: aryl hydrocarbon receptor
AID: activation-induced cytidine deaminase
APRIL: a proliferation-inducing ligand
BAFF: B cell activation factor of the TNF family
CSR: class switch recombination
CVD: cardiovascular disease
CARD9: caspase recruitment domain-containing protein 9
CKD: chronic kidney disease
DN: diabetic nephropathy
EB: Epstein-Barr
eGFR: estimated glomerular filtration rate
ESKD: end-stage kidney disease
FMT: fecal microbiota transplantation
Gd-IgA1: galactose-deficient IgA1
GALT: gut-associated lymphoid tissues
GWAS: genome-wide association studies
HCs: healthy controls
IS: Indoxyl sulfate
IgAN: IgA nephropathy
IL-6: interleukin (IL)-6
LPS: lipopolysaccharides
LIF: leukemia inhibitory factor
MUC2: mucin2
MALT: mucosa-associated lymphoid tissue
MR: mendelian randomization
NALT: nasal-associated lymphoid tissue
SCFAs: short-chain fatty acids
SNPs: single nucleotide polymorphisms
SGLT2: sodium-glucose co-transporter 2
TMAO: trimethylamine-N-oxide
TLR4: toll-like receptor-4
T2D: type 2 diabetes
VC: vascular calcification

## References

[1] Kidney Disease (2024). Improving Global Outcomes (KDIGO) CKD Work Group. KDIGO 2024 Clinical Practice Guideline for the Evaluation and Management of Chronic Kidney Disease. Kidney Int.

[2] GBD Chronic Kidney Disease Collaboration (2020). Global, regional, and national burden of chronic kidney disease, 1990-2017: a systematic analysis for the Global Burden of Disease Study 2017. Lancet.

[3] Spence JD, Urquhart BL (2022). Cerebrovascular Disease, Cardiovascular Disease, and Chronic Kidney Disease: Interplays and Influences. Curr Neurol Neurosci Rep.

[4] Canney M, Barbour SJ, Zheng Y, Coppo R, Zhang H, Liu ZH (2021). Quantifying Duration of Proteinuria Remission and Association with Clinical Outcome in IgA Nephropathy. J Am Soc Nephrol.

[5] Pitcher D, Braddon F, Hendry B, Mercer A, Osmaston K, Saleem MA (2023). Long-Term Outcomes in IgA Nephropathy. Clin J Am Soc Nephrol.

[6] Matsumoto Y, Aryal RP, Heimburg-Molinaro J, Park SS, Wever WJ, Lehoux S (2022). Identification and characterization of circulating immune complexes in IgA nephropathy. Sci Adv.

[7] Cox SN, Sallustio F, Serino G, Loverre A, Pesce F, Gigante M (2012). Activated innate immunity and the involvement of CX3CR1-fractalkine in promoting hematuria in patients with IgA nephropathy. Kidney Int.

[8] Shi N, Li N, Duan X, Niu H (2017). Interaction between the gut microbiome and mucosal immune system. Mil Med Res.

[9] Adak A, Khan MR (2019). An insight into gut microbiota and its functionalities. Cell Mol Life Sci.

[10] Gomaa EZ (2020). Human gut microbiota/microbiome in health and diseases: a review. Antonie Van Leeuwenhoek.

[11] Matsuura R, Doi K, Rabb H (2023). Acute kidney injury and distant organ dysfunction-network system analysis. Kidney Int.

[12] Packer M (2023). SGLT2 inhibitors: role in protective reprogramming of cardiac nutrient transport and metabolism. Nat Rev Cardiol.

[13] Zhang Y, Zhong W, Liu W, Wang X, Lin G, Lin J (2024). Uncovering specific taxonomic and functional alteration of gut microbiota in chronic kidney disease through 16S rRNA data. Front Cell Infect Microbiol.

[14] Wu IW, Gao SS, Chou HC, Yang HY, Chang LC, Kuo YL (2020). Integrative metagenomic and metabolomic analyses reveal severity-specific signatures of gut microbiota in chronic kidney disease. Theranostics.

[15] Wang X, Yang S, Li S, Zhao L, Hao Y, Qin J (2020). Aberrant gut microbiota alters host metabolome and impacts renal failure in humans and rodents. Gut.

[16] Hao QY, Yan J, Wei JT, Zeng YH, Feng LY, Que DD (2024). Prevotella copri promotes vascular calcification via lipopolysaccharide through activation of NF-κB signaling pathway. Gut Microbes.

[17] Martín R, Rios-Covian D, Huillet E, Auger S, Khazaal S, Bermúdez-Humarán LG (2023). Faecalibacterium: a bacterial genus with promising human health applications. FEMS Microbiol Rev.

[18] Li HB, Xu ML, Xu XD, Tang YY, Jiang HL, Li L (2022). Faecalibacterium prausnitzii Attenuates CKD via Butyrate-Renal GPR43 Axis. Circ Res.

[19] Zafar H, Saier MH Jr (2021). Gut Bacteroides species in health and disease. Gut Microbes.

[20] Huang Y, Cao J, Zhu M, Wang Z, Jin Z, Xiong Z (2024). Nontoxigenic Bacteroides fragilis: A double-edged sword. Microbiol Res.

[21] Valguarnera E, Wardenburg JB (2020). Good Gone Bad: One Toxin Away From Disease for Bacteroides fragilis. J Mol Biol.

[22] Zhou W, Wu WH, Si ZL, Liu HL, Wang H, Jiang H (2022). The gut microbe Bacteroides fragilis ameliorates renal fibrosis in mice. Nat Commun.

[23] Park NJ, Yu S, Kim DH, Yun EJ, Kim KH (2021). Characterization of BpGH16A of Bacteroides plebeius, a key enzyme initiating the depolymerization of agarose in the human gut. Appl Microbiol Biotechnol.

[24] Pei T, Zhu D, Yang S, Hu R, Wang F, Zhang J (2022). Bacteroides plebeius improves muscle wasting in chronic kidney disease by modulating the gut-renal muscle axis. J Cell Mol Med.

[25] Zhang Z, Zhao L, Wu J, Pan Y, Zhao G, Li Z (2023). The Effects of Lactobacillus johnsonii on Diseases and Its Potential Applications. Microorganisms.

[26] Miao H, Liu F, Wang YN, Yu XY, Zhuang S, Guo Y (2024). Targeting Lactobacillus johnsonii to reverse chronic kidney disease. Signal Transduct Target Ther.

[27] Zhao Q, Yu J, Hao Y, Zhou H, Hu Y, Zhang C (2023). Akkermansia muciniphila plays critical roles in host health. Crit Rev Microbiol.

[28] Zhai Q, Feng S, Arjan N, Chen W (2019). A next generation probiotic, Akkermansia muciniphila. Crit Rev Food Sci Nutr.

[29] Pei T, Hu R, Wang F, Yang S, Feng H, Li Q (2023). Akkermansia muciniphila ameliorates chronic kidney disease interstitial fibrosis via the gut-renal axis. Microb Pathog.

[30] Gleeson PJ, Benech N, Chemouny J, Metallinou E, Berthelot L, da Silva J (2024). The gut microbiota posttranslationally modifies IgA1 in autoimmune glomerulonephritis. Sci Transl Med.

[31] Fan G, Cao F, Kuang T, Yi H, Zhao C, Wang L (2023). Alterations in the gut virome are associated with type 2 diabetes and diabetic nephropathy. Gut Microbes.

[32] Mestecky J, Julian BA, Raska M (2023). IgA Nephropathy: Pleiotropic impact of Epstein-Barr virus infection on immunopathogenesis and racial incidence of the disease. Front Immunol.

[33] Zachova K, Kosztyu P, Zadrazil J, Matousovic K, Vondrak K, Hubacek P (2020). Role of Epstein-Barr Virus in Pathogenesis and Racial Distribution of IgA Nephropathy. Front Immunol.

[34] Hu J, Wei S, Gu Y, Wang Y, Feng Y, Sheng J (2022). Gut Mycobiome in Patients With Chronic Kidney Disease Was Altered and Associated With Immunological Profiles. Front Immunol.

[35] Tungsanga S, Panpetch W, Bhunyakarnjanarat T, Udompornpitak K, Katavetin P, Chancharoenthana W (2022). Uremia-Induced Gut Barrier Defect in 5/6 Nephrectomized Mice Is Worsened by Candida Administration through a Synergy of Uremic Toxin, Lipopolysaccharide, and (1➔3)-β-D-Glucan, but Is Attenuated by Lacticaseibacillus rhamnosus L34. Int J Mol Sci.

[36] Li XV, Leonardi I, Putzel GG, Semon A, Fiers WD, Kusakabe T (2022). Immune regulation by fungal strain diversity in inflammatory bowel disease. Nature.

[37] Zhuang M, Zhang X, Cai J (2024). Microbiota-gut-brain axis: interplay between microbiota, barrier function and lymphatic system. Gut Microbes.

[38] Ichihara A, Yatabe MS (2019). The (pro)renin receptor in health and disease. Nat Rev Nephrol.

[39] Lu R, Kiernan MC, Murray A, Rosner MH, Ronco C (2015). Kidney-brain crosstalk in the acute and chronic setting. Nat Rev Nephrol.

[40] Hamed SA (2019). Neurologic conditions and disorders of uremic syndrome of chronic kidney disease: presentations, causes, and treatment strategies. Expert Rev Clin Pharmacol.

[41] Bobot M, Thomas L, Moyon A, Fernandez S, McKay N, Balasse L (2020). Uremic Toxic Blood-Brain Barrier Disruption Mediated by AhR Activation Leads to Cognitive Impairment during Experimental Renal Dysfunction. J Am Soc Nephrol.

[42] Lin YT, Wu PH, Tsai YC, Hsu YL, Wang HY, Kuo MC (2019). Indoxyl Sulfate Induces Apoptosis Through Oxidative Stress and Mitogen-Activated Protein Kinase Signaling Pathway Inhibition in Human Astrocytes. J Clin Med.

[43] Hernandez L, Ward LJ, Arefin S, Ebert T, Laucyte-Cibulskiene A, Heimbürger O (2022). Blood-brain barrier and gut barrier dysfunction in chronic kidney disease with a focus on circulating biomarkers and tight junction proteins. Sci Rep.

[44] Chen X, Kong J, Pan J, Huang K, Zhou W, Diao X (2021). Kidney damage causally affects the brain cortical structure: A Mendelian randomization study. EBioMedicine.

[45] Dulam V, Katta S, Nakka VP (2024). Stroke and Distal Organ Damage: Exploring Brain-Kidney Crosstalk. Neurochem Res.

[46] Faucher Q, van der Made TK, De Lange E, Masereeuw R (2023). Blood-brain barrier perturbations by uremic toxins: Key contributors in chronic kidney disease-induced neurological disorders?. Eur J Pharm Sci.

[47] Trebicka J, Macnaughtan J, Schnabl B, Shawcross DL, Bajaj JS (2021). The microbiota in cirrhosis and its role in hepatic decompensation. J Hepatol.

[48] Pose E, Piano S, Juanola A, Ginès P (2024). Hepatorenal Syndrome in Cirrhosis. Gastroenterology.

[49] Mitrofanova A, Merscher S, Fornoni A (2023). Kidney lipid dysmetabolism and lipid droplet accumulation in chronic kidney disease. Nat Rev Nephrol.

[50] Speer T, Ridker PM, von Eckardstein A, Schunk SJ, Fliser D (2021). Lipoproteins in chronic kidney disease: from bench to bedside. Eur Heart J.

[51] Arakawa H, Kato Y (2023). Emerging Roles of Uremic Toxins and Inflammatory Cytokines in the Alteration of Hepatic Drug Disposition in Patients with Kidney Dysfunction. Drug Metab Dispos.

[52] Rosenthal SB, Bush KT, Nigam SK (2019). A Network of SLC and ABC Transporter and DME Genes Involved in Remote Sensing and Signaling in the Gut-Liver-Kidney Axis. Sci Rep.

[53] Nolin TD, Frye RF, Le P, Sadr H, Naud J, Leblond FA (2009). ESRD impairs nonrenal clearance of fexofenadine but not midazolam. J Am Soc Nephrol.

[54] Santana Machado T, Poitevin S, Paul P, McKay N, Jourde-Chiche N, Legris T (2018). Indoxyl Sulfate Upregulates Liver P-Glycoprotein Expression and Activity through Aryl Hydrocarbon Receptor Signaling. J Am Soc Nephrol.

[55] Barnes KJ, Rowland A, Polasek TM, Miners JO (2014). Inhibition of human drug-metabolising cytochrome P450 and UDP-glucuronosyltransferase enzyme activities in vitro by uremic toxins. Eur J Clin Pharmacol.

[56] Hemmati M, Kashanipoor S, Mazaheri P, Alibabaei F, Babaeizad A, Asli S (2023). Importance of gut microbiota metabolites in the development of cardiovascular diseases (CVD). Life Sci.

[57] Tan X, Liu Y, Long J, Chen S, Liao G, Wu S (2019). Trimethylamine N-Oxide Aggravates Liver Steatosis through Modulation of Bile Acid Metabolism and Inhibition of Farnesoid X Receptor Signaling in Nonalcoholic Fatty Liver Disease. Mol Nutr Food Res.

[58] Aitken AE, Morgan ET (2007). Gene-specific effects of inflammatory cytokines on cytochrome P450 2C, 2B6 and 3A4 mRNA levels in human hepatocytes. Drug Metab Dispos.

[59] Chen TK, Knicely DH, Grams ME (2019). Chronic Kidney Disease Diagnosis and Management: A Review. Jama.

[60] Huang Y, Xin W, Xiong J, Yao M, Zhang B, Zhao J (2022). The Intestinal Microbiota and Metabolites in the Gut-Kidney-Heart Axis of Chronic Kidney Disease. Front Pharmacol.

[61] Zhao J, Zhang Q, Cheng W, Dai Q, Wei Z, Guo M (2023). Heart-gut microbiota communication determines the severity of cardiac injury after myocardial ischaemia/reperfusion. Cardiovasc Res.

[62] Yang T, Richards EM, Pepine CJ, Raizada MK (2018). The gut microbiota and the brain-gut-kidney axis in hypertension and chronic kidney disease. Nat Rev Nephrol.

[63] O'Donnell JA, Zheng T, Meric G, Marques FZ (2023). The gut microbiome and hypertension. Nat Rev Nephrol.

[64] Tungsanga S, Udompornpitak K, Worasilchai J, Ratana-Aneckchai T, Wannigama DL, Katavetin P (2022). Candida Administration in 5/6 Nephrectomized Mice Enhanced Fibrosis in Internal Organs: An Impact of Lipopolysaccharide and (1→3)-β-D-Glucan from Leaky Gut. Int J Mol Sci.

[65] Szeto CC, Kwan BC, Chow KM, Kwok JS, Lai KB, Cheng PM (2015). Circulating bacterial-derived DNA fragment level is a strong predictor of cardiovascular disease in peritoneal dialysis patients. PLoS One.

[66] Szeto CC, McIntyre CW, Li PK (2018). Circulating Bacterial Fragments as Cardiovascular Risk Factors in CKD. J Am Soc Nephrol.

[67] Paladugu B, Kumar A, Parrillo JE, Der S, Osman J, Mensing J (2004). Bacterial DNA and RNA induce rat cardiac myocyte contraction depression in vitro. Shock.

[68] Han B, Zhang X, Wang L, Yuan W (2023). Dysbiosis of Gut Microbiota Contributes to Uremic Cardiomyopathy via Induction of IFNγ-Producing CD4(+) T Cells Expansion. Microbiol Spectr.

[69] Buchanan S, Combet E, Stenvinkel P, Shiels PG (2020). Klotho, Aging, and the Failing Kidney. Front Endocrinol (Lausanne).

[70] Vergani S, Muleta KG, Da Silva C, Doyle A, Kristiansen TA, Sodini S (2022). A self-sustaining layer of early-life-origin B cells drives steady-state IgA responses in the adult gut. Immunity.

[71] Zhou X, Motta F, Selmi C, Ridgway WM, Gershwin ME, Zhang W (2021). Antibody glycosylation in autoimmune diseases. Autoimmun Rev.

[72] Groza Y, Jemelkova J, Kafkova LR, Maly P, Raska M (2022). IL-6 and its role in IgA nephropathy development. Cytokine Growth Factor Rev.

[73] Jemelkova J, Stuchlova Horynova M, Kosztyu P, Zachova K, Zadrazil J, Galuszkova D (2023). GalNAc-T14 may Contribute to Production of Galactose-Deficient Immunoglobulin A1, the Main Autoantigen in IgA Nephropathy. Kidney Int Rep.

[74] Perše M, Večerić-Haler Ž (2019). The Role of IgA in the Pathogenesis of IgA Nephropathy. Int J Mol Sci.

[75] Gale DP, Molyneux K, Wimbury D, Higgins P, Levine AP, Caplin B (2017). Galactosylation of IgA1 Is Associated with Common Variation in C1GALT1. J Am Soc Nephrol.

[76] Kiryluk K, Li Y, Moldoveanu Z, Suzuki H, Reily C, Hou P (2017). GWAS for serum galactose-deficient IgA1 implicates critical genes of the O-glycosylation pathway. PLoS Genet.

[77] Wang YN, Zhou XJ, Chen P, Yu GZ, Zhang X, Hou P (2021). Interaction between GALNT12 and C1GALT1 Associates with Galactose-Deficient IgA1 and IgA Nephropathy. J Am Soc Nephrol.

[78] Bergstrom K, Liu X, Zhao Y, Gao N, Wu Q, Song K (2016). Defective Intestinal Mucin-Type O-Glycosylation Causes Spontaneous Colitis-Associated Cancer in Mice. Gastroenterology.

[79] Perez-Muñoz ME, Bergstrom K, Peng V, Schmaltz R, Jimenez-Cardona R, Marsteller N (2014). Discordance between changes in the gut microbiota and pathogenicity in a mouse model of spontaneous colitis. Gut Microbes.

[80] He JW, Zhou XJ, Li YF, Wang YN, Liu LJ, Shi SF (2021). Associations of Genetic Variants Contributing to Gut Microbiota Composition in Immunoglobin A Nephropathy. mSystems.

[81] Liang X, Zhang S, Zhang D, Hu L, Zhang L, Peng Y (2022). Metagenomics-based systematic analysis reveals that gut microbiota Gd-IgA1-associated enzymes may play a key role in IgA nephropathy. Front Mol Biosci.

[82] Zhong Z, Tan J, Tan L, Tang Y, Qiu Z, Pei G (2020). Modifications of gut microbiota are associated with the severity of IgA nephropathy in the Chinese population. Int Immunopharmacol.

[83] Tang Y, Xiao Y, He H, Zhu Y, Sun W, Hu P (2023). Aberrant Gut Microbiome Contributes to Barrier Dysfunction, Inflammation and Local Immune Responses in IgA Nephropathy. Kidney Blood Press Res.

[84] Suzuki H, Raska M, Yamada K, Moldoveanu Z, Julian BA, Wyatt RJ (2014). Cytokines alter IgA1 O-glycosylation by dysregulating C1GalT1 and ST6GalNAc-II enzymes. J Biol Chem.

[85] Yamada K, Huang ZQ, Raska M, Reily C, Anderson JC, Suzuki H (2017). Inhibition of STAT3 Signaling Reduces IgA1 Autoantigen Production in IgA Nephropathy. Kidney Int Rep.

[86] Yamada K, Huang ZQ, Raska M, Reily C, Anderson JC, Suzuki H (2020). Leukemia Inhibitory Factor Signaling Enhances Production of Galactose-Deficient IgA1 in IgA Nephropathy. Kidney Dis (Basel).

[87] Barratt J, Bailey EM, Buck KS, Mailley J, Moayyedi P, Feehally J (1999). Exaggerated systemic antibody response to mucosal Helicobacter pylori infection in IgA nephropathy. Am J Kidney Dis.

[88] Liu XZ, Zhang YM, Jia NY, Zhang H (2020). Helicobacter pylori infection is associated with elevated galactose-deficient IgA1 in IgA nephropathy. Ren Fail.

[89] Dong R, Bai M, Zhao J, Wang D, Ning X, Sun S (2020). A Comparative Study of the Gut Microbiota Associated With Immunoglobulin a Nephropathy and Membranous Nephropathy. Front Cell Infect Microbiol.

[90] Hu X, Du J, Xie Y, Huang Q, Xiao Y, Chen J (2020). Fecal microbiota characteristics of Chinese patients with primary IgA nephropathy: a cross-sectional study. BMC Nephrol.

[91] Dong Y, Chen J, Zhang Y, Wang Z, Shang J, Zhao Z (2022). Development and validation of diagnostic models for immunoglobulin A nephropathy based on gut microbes. Front Cell Infect Microbiol.

[92] Zhao J, Bai M, Ning X, Qin Y, Wang Y, Yu Z (2022). Expansion of Escherichia-Shigella in Gut Is Associated with the Onset and Response to Immunosuppressive Therapy of IgA Nephropathy. J Am Soc Nephrol.

[93] Bao WH, Tang W (2023). [Changes of gut microflora in newly diagnosed IgA nephropathy patients and its correlation with clinical risk factors]. Beijing Da Xue Xue Bao Yi Xue Ban.

[94] Shah NB, Nigwekar SU, Kalim S, Lelouvier B, Servant F, Dalal M (2021). The Gut and Blood Microbiome in IgA Nephropathy and Healthy Controls. Kidney360.

[95] Tang Y, Zhu Y, He H, Peng Y, Hu P, Wu J (2022). Gut Dysbiosis and Intestinal Barrier Dysfunction Promotes IgA Nephropathy by Increasing the Production of Gd-IgA1. Front Med (Lausanne).

[96] De Angelis M, Montemurno E, Piccolo M, Vannini L, Lauriero G, Maranzano V (2014). Microbiota and metabolome associated with immunoglobulin A nephropathy (IgAN). PLoS One.

[97] Yuan X, Qing J, Zhi W, Wu F, Yan Y, Li Y (2024). Gut and respiratory microbiota landscapes in IgA nephropathy: a cross-sectional study. Ren Fail.

[98] Gao L, Li H, Liu X, Li H, Li P, Lu W (2024). Humoral immune responses primed by the alteration of gut microbiota were associated with galactose-deficient IgA1 production in IgA nephropathy. Front Immunol.

[99] Zhu Y, He H, Sun W, Wu J, Xiao Y, Peng Y (2024). IgA nephropathy: gut microbiome regulates the production of hypoglycosilated IgA1via the TLR4 signaling pathway. Nephrol Dial Transplant.

[100] Tang Y, Xiao Y, He H, Zhu Y, Sun W, Hu P (2023). Aberrant Gut Microbiome Contributes to Barrier Dysfunction, Inflammation, and Local Immune Responses in IgA Nephropathy. Kidney Blood Press Res.

[101] Tan J, Dong L, Jiang Z, Tan L, Luo X, Pei G (2022). Probiotics ameliorate IgA nephropathy by improving gut dysbiosis and blunting NLRP3 signaling. J Transl Med.

[102] Wu H, Tang D, Zheng F, Li S, Zhang X, Yin L (2021). Identification of a novel interplay between intestinal bacteria and metabolites in Chinese patients with IgA nephropathy via integrated microbiome and metabolome approaches. Ann Transl Med.

[103] Peters BA, Qi Q, Usyk M, Daviglus ML, Cai J, Franceschini N (2023). Association of the gut microbiome with kidney function and damage in the Hispanic Community Health Study/Study of Latinos (HCHS/SOL). Gut Microbes.

[104] Federici S, Kviatcovsky D, Valdés-Mas R, Elinav E (2023). Microbiome-phage interactions in inflammatory bowel disease. Clin Microbiol Infect.

[105] Chai L, Luo Q, Cai K, Wang K, Xu B (2021). Reduced fecal short-chain fatty acids levels and the relationship with gut microbiota in IgA nephropathy. BMC Nephrol.

[106] Wu H, Tang D, Yun M, Liu H, Huang S, Yun C (2022). Metabolic Dysfunctions of Intestinal Fatty Acids and Tryptophan Reveal Immuno-Inflammatory Response Activation in IgA Nephropathy. Front Med (Lausanne).

[107] Sui W, Li L, Che W, Guimai Z, Chen J, Li W (2012). A proton nuclear magnetic resonance-based metabonomics study of metabolic profiling in immunoglobulin a nephropathy. Clinics (Sao Paulo).

[108] Jeon YH, Lee S, Kim DW, Kim S, Bae SS, Han M (2023). Serum and urine metabolomic biomarkers for predicting prognosis in patients with immunoglobulin A nephropathy. Kidney Res Clin Pract.

[109] McCarthy DD, Kujawa J, Wilson C, Papandile A, Poreci U, Porfilio EA (2011). Mice overexpressing BAFF develop a commensal flora-dependent, IgA-associated nephropathy. J Clin Invest.

[110] Chemouny JM, Gleeson PJ, Abbad L, Lauriero G, Boedec E, Le Roux K (2019). Modulation of the microbiota by oral antibiotics treats immunoglobulin A nephropathy in humanized mice. Nephrol Dial Transplant.

[111] Lauriero G, Abbad L, Vacca M, Celano G, Chemouny JM, Calasso M (2021). Fecal Microbiota Transplantation Modulates Renal Phenotype in the Humanized Mouse Model of IgA Nephropathy. Front Immunol.

[112] Di Leo V, Gleeson PJ, Sallustio F, Bounaix C, Da Silva J, Loreto G (2021). Rifaximin as a Potential Treatment for IgA Nephropathy in a Humanized Mice Model. J Pers Med.

[113] Ray N, Krieg A (2003). Oral pretreatment of mice with CpG DNA reduces susceptibility to oral or intraperitoneal challenge with virulent Listeria monocytogenes. Infection and immunity.

[114] Fukunaga M, Suriki K, Kuda T, Shikano A, Toyama A, Takahashi H (2019). Typical indigenous bacteria in the cecum of ddY mice fed a casein-beef tallow diet or whole-egg diet. J Food Biochem.

[115] Fukunaga M, Kuda T, Xia Y, Nakamura S, Takahashi H, Kimura B (2020). Detection and isolation of the typical gut indigenous bacteria from ddY mice fed a casein-beef tallow-based or egg yolk-based diet. J Food Biochem.

[116] Wang F, Li N, Ni S, Min Y, Wei K, Sun H (2023). The Effects of Specific Gut Microbiota and Metabolites on IgA Nephropathy-Based on Mendelian Randomization and Clinical Validation. Nutrients.

[117] Ren F, Jin Q, Liu T, Ren X, Zhan Y (2023). Causal effects between gut microbiota and IgA nephropathy: a bidirectional Mendelian randomization study. Front Cell Infect Microbiol.

[118] Yu S, Li X, Wang T, Li J, Li H, Xu Y (2024). B-Cell Epigenetic Modulation of IgA Response by 5-Azacytidine and IgA Nephropathy. J Am Soc Nephrol.

[119] Hu S, Bao H, Xu X, Zhou X, Qin W, Zeng C (2015). Increased miR-374b promotes cell proliferation and the production of aberrant glycosylated IgA1 in B cells of IgA nephropathy. FEBS Lett.

[120] Sallustio F, Picerno A, Cimmarusti MT, Montenegro F, Curci C, De Palma G (2023). Elevated levels of IL-6 in IgA nephropathy patients are induced by an epigenetically driven mechanism modulated by viral and bacterial RNA. Eur J Intern Med.

[121] Sharma SA, Oladejo SO, Kuang Z Chemical interplay between gut microbiota and epigenetics: Implications in circadian biology. Cell Chem Biol. 2024:S2451-9456(24)00178-8.

[122] Mostafavi Abdolmaleky H, Zhou JR (2024). Gut Microbiota Dysbiosis, Oxidative Stress, Inflammation, and Epigenetic Alterations in Metabolic Diseases. Antioxidants (Basel).

[123] Woo V, Alenghat T (2022). Epigenetic regulation by gut microbiota. Gut Microbes.

[124] Wang Z, Zhou H, Cheng F, Zhang Z, Long S (2022). miR-21 Negatively Regulates the PTEN-PI3K-Akt-mTOR Signaling Pathway in Crohn's Disease by Altering Immune Tolerance and Epithelial-Mesenchymal Transition. Discov Med.

[125] Casado-Bedmar M, Roy M, Berthet L, Hugot JP, Yang C, Manceau H (2024). Fecal let-7b and miR-21 directly modulate the intestinal microbiota, driving chronic inflammation. Gut Microbes.

[126] Bharti N, Agrawal V, Kamthan S, Prasad N, Agarwal V (2023). Blood TGF-β1 and miRNA-21-5p levels predict renal fibrosis and outcome in IgA nephropathy. Int Urol Nephrol.

[127] Serino G, Pesce F, Sallustio F, De Palma G, Cox SN, Curci C (2016). In a retrospective international study, circulating miR-148b and let-7b were found to be serum markers for detecting primary IgA nephropathy. Kidney Int.

[128] Casali P, Li S, Morales G, Daw CC, Chupp DP, Fisher AD (2021). Epigenetic Modulation of Class-Switch DNA Recombination to IgA by miR-146a Through Downregulation of Smad2, Smad3 and Smad4. Front Immunol.

[129] Schroeder BO (2019). Fight them or feed them: how the intestinal mucus layer manages the gut microbiota. Gastroenterol Rep (Oxf).

[130] Martens EC, Neumann M, Desai MS (2018). Interactions of commensal and pathogenic microorganisms with the intestinal mucosal barrier. Nat Rev Microbiol.

[131] Harrison CA, Laubitz D, Ohland CL, Midura-Kiela MT, Patil K, Besselsen DG (2018). Microbial dysbiosis associated with impaired intestinal Na(+)/H(+) exchange accelerates and exacerbates colitis in ex-germ free mice. Mucosal Immunol.

[132] Taleb S (2019). Tryptophan Dietary Impacts Gut Barrier and Metabolic Diseases. Front Immunol.

[133] Ouwerkerk JP, de Vos WM, Belzer C (2013). Glycobiome: bacteria and mucus at the epithelial interface. Best Pract Res Clin Gastroenterol.

[134] Glover JS, Ticer TD, Engevik MA (2022). Characterizing the mucin-degrading capacity of the human gut microbiota. Sci Rep.

[135] Gamage H, Chong RWW, Bucio-Noble D, Kautto L, Hardikar AA, Ball MS (2020). Changes in dietary fiber intake in mice reveal associations between colonic mucin O-glycosylation and specific gut bacteria. Gut Microbes.

[136] Bell A, Brunt J, Crost E, Vaux L, Nepravishta R, Owen CD (2019). Elucidation of a sialic acid metabolism pathway in mucus-foraging Ruminococcus gnavus unravels mechanisms of bacterial adaptation to the gut. Nat Microbiol.

[137] Rostoker G, Wirquin V, Terzidis H, Petit-Phar M, Chaumette M, Delchier J (1993). Mucosal immunity in primary glomerulonephritis. III. Study of intestinal permeability. Nephron.

[138] Buck KS, Smith AC, Molyneux K, El-Barbary H, Feehally J, Barratt J (2008). B-cell O-galactosyltransferase activity, and expression of O-glycosylation genes in bone marrow in IgA nephropathy. Kidney Int.

[139] Dotz V, Visconti A, Lomax-Browne HJ, Clerc F, Hipgrave Ederveen AL, Medjeral-Thomas NR (2021). O- and N-Glycosylation of Serum Immunoglobulin A is Associated with IgA Nephropathy and Glomerular Function. J Am Soc Nephrol.

[140] Bondareva M, Budzinski L, Durek P, Witkowski M, Angermair S, Ninnemann J (2023). Cross-regulation of antibody responses against the SARS-CoV-2 Spike protein and commensal microbiota via molecular mimicry. Cell Host Microbe.

[141] Shahrizaila N, Lehmann HC, Kuwabara S (2021). Guillain-Barré syndrome. Lancet.

[142] Vitale AM, Paladino L, Caruso Bavisotto C, Barone R, Rappa F, Conway de Macario E (2024). Interplay between the Chaperone System and Gut Microbiota Dysbiosis in Systemic Lupus Erythematosus Pathogenesis: Is Molecular Mimicry the Missing Link between Those Two Factors?. Int J Mol Sci.

[143] Chen Q, Jiang H, Ding R, Zhong J, Li L, Wan J (2023). Cell-type-specific molecular characterization of cells from circulation and kidney in IgA nephropathy with nephrotic syndrome. Front Immunol.

[144] Lavine N, Ohayon A, Mahroum N (2022). Renal autoimmunity: The role of bacterial and viral infections, an extensive review. Autoimmun Rev.

[145] Nihei Y, Kitamura D Pathogenesis of IgA nephropathy as a tissue-specific autoimmune disease. Int Immunol. 2024: dxae047.

[146] Higashiyama M, Haniuda K, Nihei Y, Kazuno S, Kikkawa M, Miura Y (2024). Oral bacteria induce IgA autoantibodies against a mesangial protein in IgA nephropathy model mice. Life Sci Alliance.

[147] Nihei Y, Haniuda K, Higashiyama M, Asami S, Iwasaki H, Fukao Y (2023). Identification of IgA autoantibodies targeting mesangial cells redefines the pathogenesis of IgA nephropathy. Sci Adv.

[148] Kim C (2021). Control of lymphocyte functions by gut microbiota-derived short-chain fatty acids. Cellular & molecular immunology.

[149] Burger-van Paassen N, Vincent A, Puiman P, van der Sluis M, Bouma J, Boehm G (2009). The regulation of intestinal mucin MUC2 expression by short-chain fatty acids: implications for epithelial protection. The Biochemical journal.

[150] Isobe J, Maeda S, Obata Y, Iizuka K, Nakamura Y, Fujimura Y (2020). Commensal-bacteria-derived butyrate promotes the T-cell-independent IgA response in the colon. Int Immunol.

[151] Deleu S, Machiels K, Raes J, Verbeke K, Vermeire S (2021). Short chain fatty acids and its producing organisms: An overlooked therapy for IBD?. EBioMedicine.

[152] Lavelle A, Sokol H (2020). Gut microbiota-derived metabolites as key actors in inflammatory bowel disease. Nat Rev Gastroenterol Hepatol.

[153] Su X, Gao Y, Yang R (2022). Gut Microbiota-Derived Tryptophan Metabolites Maintain Gut and Systemic Homeostasis. Cells.

[154] Dodd D, Spitzer MH, Van Treuren W, Merrill BD, Hryckowian AJ, Higginbottom SK (2017). A gut bacterial pathway metabolizes aromatic amino acids into nine circulating metabolites. Nature.

[155] Fagarasan S, Muramatsu M, Suzuki K, Nagaoka H, Hiai H, Honjo T (2002). Critical roles of activation-induced cytidine deaminase in the homeostasis of gut flora. Science.

[156] Sterlin D, Fadlallah J, Slack E, Gorochov G (2020). The antibody/microbiota interface in health and disease. Mucosal Immunol.

[157] Yanagibashi T, Hosono A, Oyama A, Tsuda M, Hachimura S, Takahashi Y (2009). Bacteroides induce higher IgA production than Lactobacillus by increasing activation-induced cytidine deaminase expression in B cells in murine Peyer's patches. Biosci Biotechnol Biochem.

[158] Yang C, Mogno I, Contijoch EJ, Borgerding JN, Aggarwala V, Li Z (2020). Fecal IgA Levels Are Determined by Strain-Level Differences in Bacteroides ovatus and Are Modifiable by Gut Microbiota Manipulation. Cell Host Microbe.

[159] Tan J, Ni D, Taitz J, Pinget GV, Read M, Senior A (2022). Dietary protein increases T-cell-independent sIgA production through changes in gut microbiota-derived extracellular vesicles. Nat Commun.

[160] Vitari N, Singh S, Tao J, Truitt B, Kolli U, Jalodia R (2024). Morphine-induced intestinal microbial dysbiosis drives TLR-dependent IgA targeting of gram-positive bacteria and upregulation of CD11b and TLR2 on a sub-population of IgA(+) B cells. Gut Microbes.

[161] Kubinak JL, Petersen C, Stephens WZ, Soto R, Bake E, O'Connell RM (2015). MyD88 signaling in T cells directs IgA-mediated control of the microbiota to promote health. Cell Host Microbe.

[162] Kinashi Y, Tanaka K, Kimura S, Hirota M, Komiyama S, Shindo T (2024). Intestinal epithelium dysfunctions cause IgA deposition in the kidney glomeruli of intestine-specific Ap1m2-deficient mice. EBioMedicine.

[163] Hedblom GA, Reiland HA, Sylte MJ, Johnson TJ, Baumler DJ (2018). Segmented Filamentous Bacteria - Metabolism Meets Immunity. Front Microbiol.

[164] Roberts I, Cook H, Troyanov S, Alpers C, Amore A, Barratt J (2009). The Oxford classification of IgA nephropathy: pathology definitions, correlations, and reproducibility. Kidney international.

[165] Soares M, Roberts I (2017). IgA nephropathy: an update. Current opinion in nephrology and hypertension.

[166] Barbour S, Coppo R, Zhang H, Liu Z, Suzuki Y, Matsuzaki K (2019). Evaluating a New International Risk-Prediction Tool in IgA Nephropathy. JAMA internal medicine.

[167] Kidney Int RepErratum to Zhang Y, Guo L, Wang Z, Wang J, Er L, Barbour SJ, Trimarchi H, Lv J, Zhang H (2021). "External Validation of International Risk-Prediction Models of IgA Nephropathy in an Asian-Caucasian Cohort.". 2020;5:1753-1763. Kidney international reports.

[168] Wan F, Wang H, Wang M, Lv J, Zhao M, Zhang H (2022). Sustained release of Lactobacillus casei cell wall extract can induce a continuous and stable IgA deposition model. The Journal of pathology.

[169] Zhou N, Shen Y, Fan L, Sun Q, Huang C, Hao J (2020). The Characteristics of Intestinal-Barrier Damage in Rats With IgA Nephropathy. The American journal of the medical sciences.

[170] Peng S, Zeng H, Fu A, Chen X, Zhu Q (2013). Effects of rhein on intestinal epithelial tight junction in IgA nephropathy. World journal of gastroenterology.

[171] Li Y, Wan S, Liu J, Huang Y, Jiang L (2024). Causal relationship between dietary intake and IgA nephropathy: a Mendelian randomization study. Front Nutr.

[172] Woodrow G, Innes A, Boyd SM, Burden RP (1993). A case of IgA nephropathy with coeliac disease responding to a gluten-free diet. Nephrol Dial Transplant.

[173] Joshi S, Kalantar-Zadeh K, Chauveau P, Carrero JJ (2023). Risks and Benefits of Different Dietary Patterns in CKD. Am J Kidney Dis.

[174] Chemouny J, Gleeson P, Abbad L, Lauriero G, Boedec E, Le Roux K (2019). Modulation of the microbiota by oral antibiotics treats immunoglobulin A nephropathy in humanized mice. Nephrology, dialysis, transplantation: official publication of the European Dialysis and Transplant Association - European Renal Association.

[175] Mazziotta C, Tognon M, Martini F, Torreggiani E, Rotondo JC (2023). Probiotics Mechanism of Action on Immune Cells and Beneficial Effects on Human Health. Cells.

[176] Kim KS (2022). Regulation of T cell repertoires by commensal microbiota. Front Cell Infect Microbiol.

[177] Smerud HK, Bárány P, Lindström K, Fernström A, Sandell A, Påhlsson P (2011). New treatment for IgA nephropathy: enteric budesonide targeted to the ileocecal region ameliorates proteinuria. Nephrol Dial Transplant.

[178] Liao J, Zhou Y, Xu X, Huang K, Chen P, Wu Y (2022). Current knowledge of targeted-release budesonide in immunoglobulin A nephropathy: A comprehensive review. Front Immunol.

[179] Lafayette R, Kristensen J, Stone A, Floege J, Tesař V, Trimarchi H (2023). Efficacy and safety of a targeted-release formulation of budesonide in patients with primary IgA nephropathy (NefIgArd): 2-year results from a randomised phase 3 trial. Lancet.

[180] Zachova K, Jemelkova J, Kosztyu P, Ohyama Y, Takahashi K, Zadrazil J (2022). Galactose-Deficient IgA1 B cells in the Circulation of IgA Nephropathy Patients Carry Preferentially Lambda Light Chains and Mucosal Homing Receptors. Journal of the American Society of Nephrology: JASN.

[181] Liu LJ, Yang YZ, Shi SF, Bao YF, Yang C, Zhu SN (2019). Effects of Hydroxychloroquine on Proteinuria in IgA Nephropathy: A Randomized Controlled Trial. Am J Kidney Dis.

[182] Wheeler DC, Toto RD, Stefánsson BV, Jongs N, Chertow GM, Greene T (2021). A pre-specified analysis of the DAPA-CKD trial demonstrates the effects of dapagliflozin on major adverse kidney events in patients with IgA nephropathy. Kidney Int.

[183] Impact of diabetes on the effects of sodium glucose co-transporter-2 inhibitors on kidney outcomes (2022). collaborative meta-analysis of large placebo-controlled trials. Lancet.

[184] El Karoui K, Fervenza FC, De Vriese AS (2024). Treatment of IgA Nephropathy: A Rapidly Evolving Field. J Am Soc Nephrol.

[185] Billing AM, Kim YC, Gullaksen S, Schrage B, Raabe J, Hutzfeldt A (2024). Metabolic Communication by SGLT2 Inhibition. Circulation.

[186] Deng L, Yang Y, Xu G (2022). Empagliflozin ameliorates type 2 diabetes mellitus-related diabetic nephropathy via altering the gut microbiota. Biochim Biophys Acta Mol Cell Biol Lipids.

[187] Wortelboer K, Nieuwdorp M, Herrema H (2019). Fecal microbiota transplantation beyond Clostridioides difficile infections. EBioMedicine.

[188] Zhao J, Bai M, Yang X, Wang Y, Li R, Sun S (2021). Alleviation of refractory IgA nephropathy by intensive fecal microbiota transplantation: the first case reports. Ren Fail.

[189] Zhi W, Song W, Abdi Saed Y, Wang Y, Li Y (2022). Fecal Capsule as a Therapeutic Strategy in IgA Nephropathy: A Brief Report. Front Med (Lausanne).

[190] Zhi W, Li A, Wang Q, Yuan X, Qing J, Zhang C (2024). Safety and efficacy assessment of fecal microbiota transplantation as an adjunctive treatment for IgA nephropathy: an exploratory clinical trial. Sci Rep.

[191] He L, Zhou X, Liu Y, Zhou L, Li F (2022). Fecal miR-142a-3p from dextran sulfate sodium-challenge recovered mice prevents colitis by promoting the growth of Lactobacillus reuteri. Mol Ther.

[192] Sugurmar ANK, Mohd R, Shah SA, Neoh HM, Cader RA (2021). Gut microbiota in Immunoglobulin A Nephropathy: a Malaysian Perspective. BMC Nephrol.

[193] Cai F, Zhou C, Jiao N, Liang X, Ye Z, Chen W (2023). Systematic Microbiome Dysbiosis Is Associated with IgA Nephropathy. Microbiol Spectr.

[194] Kano T, Suzuki H, Makita Y, Fukao Y, Suzuki Y (2021). Nasal-associated lymphoid tissue is the major induction site for nephritogenic IgA in murine IgA nephropathy. Kidney Int.

[195] Currie EG, Coburn B, Porfilio EA, Lam P, Rojas OL, Novak J (2022). Immunoglobulin A nephropathy is characterized by anticommensal humoral immune responses. JCI Insight.

[196] Xie X, Li J, Liu P, Wang M, Gao L, Wan F (2022). Chimeric Fusion between Clostridium Ramosum IgA Protease and IgG Fc Provides Long-Lasting Clearance of IgA Deposits in Mouse Models of IgA Nephropathy. J Am Soc Nephrol.

[197] Scott SA, Fu J, Chang PV (2024). Dopamine receptor D2 confers colonization resistance via microbial metabolites. Nature.

[198] Song X, Sun X, Oh SF, Wu M, Zhang Y, Zheng W (2020). Microbial bile acid metabolites modulate gut RORγ(+) regulatory T cell homeostasis. Nature.

[199] Bousbaine D, Fisch LI, London M, Bhagchandani P, Rezende de Castro TB, Mimee M (2022). A conserved Bacteroidetes antigen induces anti-inflammatory intestinal T lymphocytes. Science.

[200] Gao J, Zhao X, Hu S, Huang Z, Hu M, Jin S (2022). Gut microbial DL-endopeptidase alleviates Crohn's disease via the NOD2 pathway. Cell Host Microbe.

